# Telomeric TART elements target the piRNA machinery in *Drosophila*

**DOI:** 10.1371/journal.pbio.3000689

**Published:** 2020-12-21

**Authors:** Christopher E. Ellison, Meenakshi S. Kagda, Weihuan Cao

**Affiliations:** Department of Genetics, Human Genetics Institute of New Jersey, Rutgers, The State University of New Jersey, Piscataway, New Jersey, United States of America; Institute of Molecular Biology, GERMANY

## Abstract

Coevolution between transposable elements (TEs) and their hosts can be antagonistic, where TEs evolve to avoid silencing and the host responds by reestablishing TE suppression, or mutualistic, where TEs are co-opted to benefit their host. The *TART-A* TE functions as an important component of *Drosophila* telomeres but has also reportedly inserted into the *Drosophila melanogaster* nuclear export factor gene *nxf2*. We find that, rather than inserting into *nxf2*, *TART-A* has actually captured a portion of *nxf2* sequence. We show that *TART-A* produces abundant Piwi-interacting small RNAs (piRNAs), some of which are antisense to the *nxf2* transcript, and that the *TART*-like region of *nxf2* is evolving rapidly. Furthermore, in *D*. *melanogaster*, *TART-A* is present at higher copy numbers, and *nxf2* shows reduced expression, compared to the closely related species *Drosophila simulans*. We propose that capturing *nxf2* sequence allowed *TART-A* to target the *nxf2* gene for piRNA-mediated repression and that these 2 elements are engaged in antagonistic coevolution despite the fact that *TART-A* is serving a critical role for its host genome.

## Abstract 2

Co-evolution between transposable elements (TEs) and their hosts can be antagonistic, where TEs evolve to avoid silencing and the host responds by re-establishing TE suppression, or mutualistic, where TEs are co-opted to benefit their host. This study shows that a specialized Drosophila retrotransposon that functions as a telomere has captured a portion of a host piRNA gene which may allow it to evade silencing.

## Introduction

Transposable elements (TEs) must replicate faster than their host to avoid extinction. The vast majority of new TE insertions derived from this replicative activity are deleterious to their host: They can disrupt and/or silence protein-coding genes and lead to chromosome rearrangements [1–3]. In response to the mutational burden imposed by TEs, TE hosts have evolved elaborate genome surveillance mechanisms to identify and target TEs for suppression.

One of the most well-known genome defense pathways in metazoan species involves the production of Piwi-interacting small RNAs, also known as piRNAs [4]. PiRNA precursors are produced from piRNA clusters, which are located in heterochromatin and contain fragments of many families of TEs, whose insertions have accumulated in these regions. These precursors are processed into phased piRNAs, which use sequence homology to guide Piwi proteins to complementary transcripts produced by active TEs [4,5]. Piwi proteins induce posttranscriptional silencing through cleavage of the TE transcript. The sense-strand cleavage product of the TE transcript can then aid in processing piRNA precursors though a process known as the ping-pong cycle, which amplifies the silencing signal [4,5]. Alternatively, the cleaved transcript can be processed by the endonuclease Zucchini into additional phased piRNAs starting from the cleavage site and proceeding in the 3′ direction.

Several recent studies have identified a novel protein complex in *Drosophila* that connects the piRNA-mediated targeting of mRNAs by Piwi with the establishment of cellular heterochromatin [6–9]. This complex contains a heterodimer consisting of Nxf2 and Nxt1 and Panoramix [6–9]. Nxf2 belongs to a family of nuclear export proteins, yet has lost the ability to export RNA and instead plays a specialized role in the piRNA pathway [6–9]. Nxf2 interacts with Piwi-targeted transcripts, while Panoramix likely recruits Lsd1 to demethylate H3K4me2 and SetDB1 to establish H3K9me3 [6–9] Interestingly, 2 paralogs of Nxf2, Nxf1, and Nxf3 play an important role in piRNA precursor export: Nxf1 exports flamenco piRNA precursors [10,11], and Nxf3 exports germline piRNA precursors [12,13], which raises the possibility that the nuclear export factor gene family may have diversified in response to TE–host conflict [6].

The ubiquity of active TEs suggests that host silencing mechanisms are not completely effective, which may be due to the fact that selection for complete TE repression is relatively weak [14–16] or because the TE and its host genome are involved in an evolutionary “arms race” where TEs are continuously evolving novel means to avoid host silencing and the host genome is constantly reestablishing TE suppression [17]. On the host side, many TE silencing components have been shown to be evolving rapidly under positive selection [18–26], in agreement with ongoing host–TE conflict.

On the transposon side, a TE can mount a counter-defense by silencing or blocking host factors [27–29] or simply evade host silencing by replicating in permissive cells [30] or cloaking themselves in virus-like particles [31]. However, there are surprisingly few examples of any of these strategies [32]. In fact, there is some evidence that, rather than an evolutionary arms race, the rapid evolution of host silencing genes is related to avoiding gene silencing due to off-target effects (i.e., piRNA autoimmunity [33,34]) and/or coevolution with viruses (reviewed in [32]).

While there are currently only a few examples of TE counter-defense strategies, there are many examples of TEs being co-opted by their host genome for its own advantage (see reviews [32,35–38]). TEs can disperse regulatory sequences across the genome [39–46] and have been co-opted as a source of host genes and noncoding RNAs [37,38,47–49]. TEs can also act as structural components of the genome. There is evidence that TEs may play a role in centromere specification in a variety of species [50–52], and in *Drosophila*, which lacks telomerase, specific TEs serve as telomeres by replicating to chromosome ends [53,54].

In *Drosophila melanogaster*, 3 related non-long terminal repeat (non-LTR) retrotransposons occupy the telomeres: *HeT-A*, *TAHRE*, and *TART*, which are often abbreviated as HTT elements [53,55–57]. These elements belong to the Jockey clade of Long Interspersed Nuclear Elements (LINEs), which contain open reading frames for gag (ORF1) and an endonuclease/reverse transcriptase protein (ORF2, lost in *HeT-A*) [58,59]. These elements form head-to-tail arrays at the chromosome ends, and their replication solves the chromosome “end-shortening” problem without the need for telomerase [60].

These telomeric elements represent a unique case of TE domestication. They serve a critical role for their host genome, yet they are still active elements, capable of causing mutational damage if their activity is left unchecked [61–63]. All 3 elements have been shown to produce abundant piRNAs and RNA interference (RNAi) knockdown and/or mutation of piRNA pathway components, including *nxf2*, leads to their up-regulation [6–9,62,64,65], consistent with the host genome acting to constrain their activity and raising the possibility that, despite being domesticated, these elements are still in conflict with their host [66]. There are multiple lines of evidence that this is indeed the case: The protein components of *Drosophila* telomeres are rapidly evolving under positive selection, potentially due to a role in preventing the HTT elements from overproliferation [66]. There is a high rate of gain and loss of HTT lineages within the melanogaster species group [67], and there is dramatic variation in telomere length among strains from the *Drosophila* Genetic Reference Panel (DGRP) [68]. These observations are more consistent with evolution under conflict rather than a stable symbiosis [67]. Furthermore, the nucleotide sequence of the HTT elements evolves extremely rapidly, especially in their unusually long 3′ UTRs [69,70]. Within *D*. *melanogaster*, 3 *TART* subfamilies have been identified which contain completely different 3′ UTRs, and which are known as *TART-A*, *TART-B*, and *TART-C* [57].

In this study, we have characterized the presence of sequence within the coding region of the *D*. *melanogaster nxf2* gene that was previously annotated as an insertion of the *TART-A* transposon [71]. We find that the shared homology between *TART-A* and *nxf2* is actually the result of *TART-A* acquiring a portion of the *nxf2* gene, rather than the *nxf2* gene gaining a *TART-A* insertion. Our findings support a model where *TART-A* produces antisense piRNAs that target *nxf2* for suppression as a counter-defense strategy in response to host silencing. We identified *nxf2* cleavage products from degradome sequencing (degradome-seq) data that are consistent with Aub-directed cleavage of *nxf2* transcripts and we find that, across the DGRP, *TART-A* copy number is negatively correlated with *nxf2* expression, and *nxf2* piRNA production is positively correlated with *TART-A* piRNA production. Furthermore, the *D*. *melanogaster nxf2* sequence is evolving rapidly in the region of shared homology with *TART-A*, and *TART-A* insertions are more abundant in *D*. *melanogaster* compared to *Drosophila simulans*. Our findings suggest that TEs can selfishly manipulate host silencing pathways in order to increase their own copy number and that a single TE family can benefit, as well as antagonize, its host genome.

## Results

### The *TART*-like region of *nxf2* is conserved across the melanogaster group

**Fig 1:**
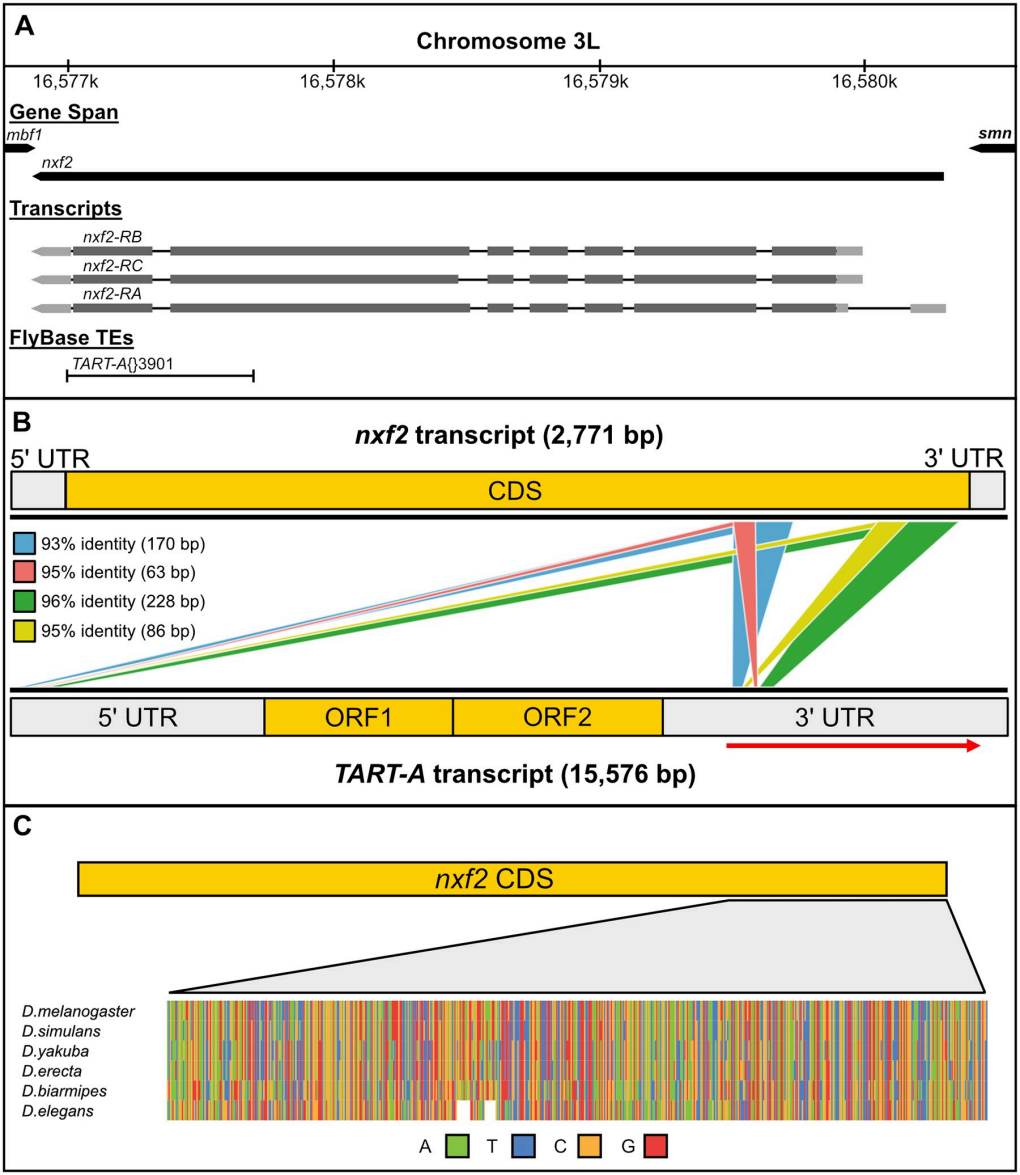
Shared homology between the *D*. *melanogaster nxf2* gene and the *TART-A* TE. (A) Gene, transcript, and TE annotations from FlyBase showing the *nxf2* gene models along with the annotated *TART-A* TE insertion. Note that the *TART-A* annotation overlaps the 3′ CDS of *nxf2*. (B) BLAST hits between the RepBase *TART-A* sequence and the *nxf2* transcript. Each colored box represents a single BLAST alignment. The 5′ UTR of *TART-A* is copied from a portion of the 3′ UTR during replication. The homology between *nxf2* and the *TART-A* 3′ UTR is therefore mirrored in the 5′ UTR. The red arrow shows the region of the 3′ UTR that the 5′ UTR is copied from. (C). A zoomed-out multiple sequence alignment of *nxf2* orthologs for 6 species from the melanogaster species group shows that the *TART-like* region of *nxf2* is present in all 6 species. The actual alignment can be found in S1 Data. CDS, coding sequence; ORF, open reading frame; TE, transposable element.

It was previously reported that the homology between *nxf2* and *TART-A* is due to an insertion of the *TART-A* TE in the *nxf2* gene that became fixed in the ancestor of *D*. *melanogaster* and *D*. *simulans* [71] (Fig 1A). To investigate the homology between these elements in more detail, we used BLAST [72] to search the *nxf2* transcript against the *TART-A* RepBase sequence, which was derived from a full-length *TART-A* element cloned from the *iso1 D*. *melanogaster* reference strain [73]. There are 4 regions of homology between *nxf2* and the 3′ UTR of *TART-A* that lie within a 700-bp segment of *nxf2*. These regions are between 63 bp and 228 bp in length and 93% to 96% sequence identity (Fig 1B). The 5′ UTR of *TART-A* is copied from a portion of the 3′ UTR during reverse transcription [74], which means that the *nxf2*-like region in the 3′ UTR is therefore mirrored in the 5′ UTR as well (Fig 1B).

To investigate the evolutionary origin of the homology between *nxf2* and *TART-A*, we identified *nxf2* orthologs in *D*. *simulans*, *Drosophila yakuba*, *Drosophila erecta*, *Drosophila biarmipes*, and *Drosophila elegans*. The *TART*-like region of *nxf2* is clearly present in all 6 of these species. Therefore, if this portion of the *nxf2* gene was derived from an insertion of a *TART-A* element, the most recent time point at which the insertion could have occurred is in the common ancestor of the melanogaster group, approximately 15 million years ago [75] (Fig 1C, S1 Data). At the nucleotide level, there is only weak homology between *nxf2* coding sequence (CDS) and transcripts from more distantly related *Drosophila* species, such as *Drosophila pseudoobscura*. However, at the peptide level, the carboxyl-terminal region of Nxf2, which was thought to be derived from *TART-A*, is actually conserved across *Drosophila*, from *D*. *melanogaster* to *Drosophila virilis* (S1 Fig), suggesting that, if a *TART-A* element did insert into the *nxf2* gene, it was not a recent event.

### A portion of *nxf2* was captured by the *D*. *melanogaster TART-A* element

**Fig 2:**
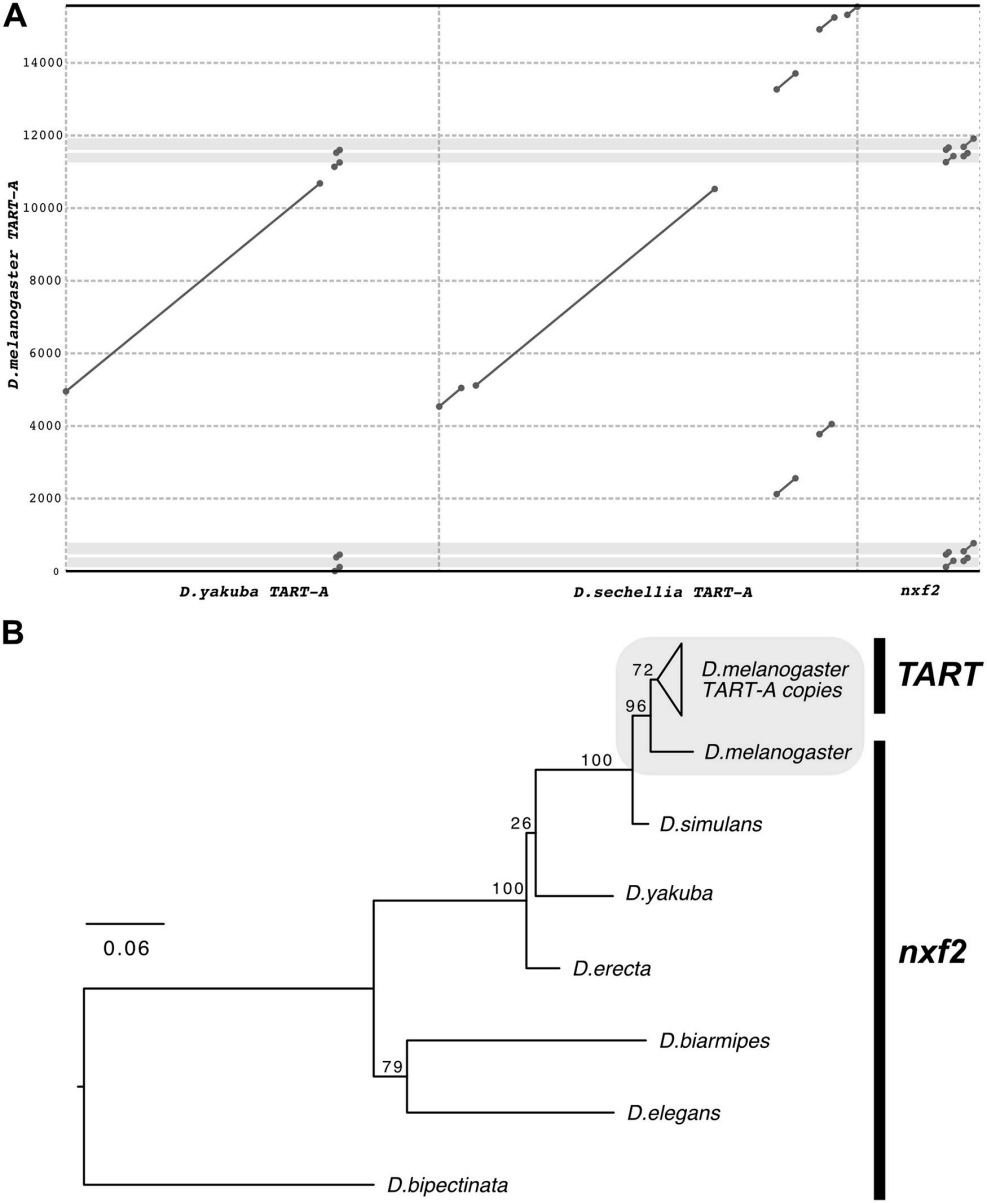
The *TART-A*/*nxf2* homology is unique to *D*. *melanogaster*. (A) Dotplot comparing *D*. *melanogaster TART-A* to its homologs in *D*. *yakuba* and *D*. *sechellia*. The diagonal lines denote regions of homology, while the light gray boxes show the location of the *nxf2*-like sequence in the *D*. *melanogaster TART-A*. Neither the *D*. *yakuba* nor the *D*. *sechellia TART-A* sequences contain *nxf2*-like sequence. However, the regions directly flanking the *nxf2*-like sequence in *D*. *melanogaster* are also present in *D*. *yakuba* (see S2 Fig for magnified view). Underlying data can be found in S2 Data. (B) Gene tree showing relative age of shared homology. We aligned the *nxf2*-like sequences from 9 copies of *TART-A* in the *D*. *melanogaster* reference genome to the *nxf2* transcripts from 6 *Drosophila* species and inferred a maximum likelihood phylogeny using RAxML. *D*. *melanogaster nxf2* is most closely related to the *nxf2*-like sequences present in the *D*. *melanogaster TART-A* copies, suggesting the shared homology occurred after the divergence between *D*. *melanogaster* and *D*. *simulans*.

If an ancestral *TART-A* element was inserted into the *nxf2* gene in the common ancestor of the melanogaster group, the shared homology between *nxf2* and *TART-A* should be present in most, if not all, extant species in the group. To test this prediction, we obtained the sequences for previously identified *TART-A* homologs from *D*. *yakuba* and *Drosophila sechellia* [59,69]. We aligned these sequences to *D*. *melanogaster TART-A* and found that the *TART-A* region that shares homology with the *nxf2* gene is only present in the *D*. *melanogaster TART-A* sequence (Fig 2A, S2 and S3 Figs, S2 Data).

We identified 9 *TART-A* elements (5 full length and 4 fragments) in the *D*. *melanogaster* reference genome assembly that contain the *nxf2*-like sequence. We added these 9 sequences to the multiple sequence alignment in Fig 1C and inferred a maximum likelihood phylogeny in order to better understand the evolutionary history of the *nxf2*/*TART* shared homology (Fig 2B, S3 Data). The youngest node in the phylogeny represents the split between the *D*. *melanogaster nxf2* and *TART-A* elements, suggesting that the event leading to the shared homology between these sequences occurred relatively recently, which is consistent with the high degree of sequence similarity between the *D*. *melanogaster TART-A* and *nxf2* subsequences. Based on these results, we conclude that the *nxf2/TART-A* shared homology is much more likely to have arisen via the recent acquisition of *nxf2* sequence by *TART-A* after the split of *D*. *melanogaster* from *D*. *simulans*/*sechellia*, rather than an insertion of *TART-A* into the *nxf2* gene. The mechanism by which *TART-A* could have acquired a portion of *nxf2* is not clear; however, one possibility is via transduction, a process where genomic regions flanking a TE insertion can be incorporated into the TE itself due to aberrant retrotransposition [76,77].

### The *nxf2*-captured region is likely to be fixed in *D*. *melanogaster*

We next sought to determine whether the *nxf2*-like *TART-A* variant is present in all dispersed copies of *TART-A* in *D*. *melanogaster*. We compared the RepBase (strain *iso1*) *TART-A* sequence to other *TART-A* sequences present in GenBank that included the 3′ UTR. We found 2 additional sequences: 1 cloned from the strain A4-4 and another cloned from the strain Oregon-R, both of which contained the *nxf2*-like region. We then searched the RepBase *TART-A* sequence against the *D*. *melanogaster* reference genome assembly plus long-read assemblies of 16 other *D*. *melanogaster* strains [78,79] (see Methods). We identified a total of 71 *TART-A* sequences that included at least a portion of the 3′ UTR. All 71 sequences also included the *nxf2*-like region (S4 Fig).

In order to survey additional strains, we next used a coverage-based approach along with Illumina (San Diego, California, United States of America) data from the DGRP [80,81]. We aligned Illumina reads to the *TART-A* RepBase sequence and compared sequencing coverage for the *nxf2*-like region to both the upstream and downstream flanking regions. We divided read coverage for these regions by each strain’s median coverage of *TART-A* ORF1 and ORF2 to control for copy number differences between strains. If the *nxf2*-like region is only present in some *TART-A* elements and missing from others, we would expect that coverage of this region should be lower than the 2 flanking regions. Across 151 DGRP strains, we found that the coverage of the *nxf2*-like region was lower than the coverage of the upstream region but similar to the coverage of the downstream region (S5 Fig). There are only 6 individuals where the coverage of the *nxf2*-like region is lower than both flanking regions. In these cases, the difference between the coverage of the *nxf2*-like region and the downstream region is very small (mean reduction of 6.3%). This pattern is not consistent with polymorphism, but rather, truncation of the 5′ UTR, which has previously been described for *TART* [74]. Because the *nxf2*-like sequence is present in both UTRs, truncation of the 5′ UTR, which is fairly common, should reduce coverage of the *nxf2*-like region by as much as 50% compared to the upstream region, which is not present in the 5′ UTR (Fig 1B). We observed a reduction in coverage of approximately 30%, consistent with a mixture of *TART-A* copies, some with truncated 5′ UTRs and some without.

### The *nxf2* gene plays a role in suppressing the activity of *D*. *melanogaster* telomeric elements

**Fig 3:**
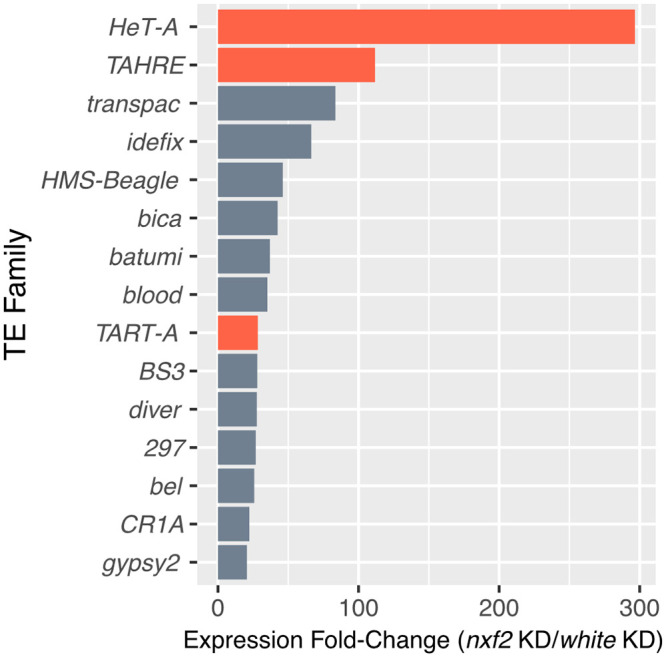
RNAi knockdown of *nxf2* leads to strong up-regulation of HTT elements. We examined TE expression profiles using RNA-seq of total RNA from ovaries in an *nxf2* knockdown versus a control knockdown of the *white* gene. We found that a variety of TEs show increased expression in the *nxf2* knockdown (see S6 Fig for all TEs); however, the 3 telomeric HTT elements (red bars) are among the top 10 most highly up-regulated TEs. Underlying data can be found in S2 Data. HTT, *HeT-A*, *TAHRE*, and *TART*; RNAi, RNA interference; RNA-seq, RNA sequencing; TE, transposable element.

Nxf2 is part of an evolutionarily conserved gene family with functions related to export of RNA from the nucleus [82]. In *Drosophila*, there are 4 nuclear export factor paralogs: *nxf1* is involved in the export of mRNAs and flamenco piRNA precursors from the nucleus [10,11], while *nxf3* plays a more specialized role in germline piRNA precursor export [12,13]. *Nxf4* shows testes specific expression; however, its exact function remains unknown. The *nxf2* gene was identified as a member of the germline piRNA pathway via an RNAi screen [10,11], and more recently, several studies have independently shown that Nxf2 is involved in the co-transcriptional silencing of transposons as part of a complex with Nxt1 and Panoramix [6–9]. Batki and colleagues reported TE derepression in a *nxf2* null mutant, including an approximately 80-fold increase in *TART-A* expression. To confirm the involvement of *nxf2* in the suppression of *TART-A*, we used a short hairpin RNA (shRNA) from the *Drosophila* Transgenic RNAi Project (TRiP) with a nos-GAL4 driver to target and knockdown expression of *nxf2* in the ovaries. The *nanos* promoter drives Gal4 expression in germline stem cells and starting in stage 5 of oogenesis, with weak expression in young egg chambers [83]. We sequenced total RNA from the *nxf2* knockdown and a control knockdown of the *white* gene. We observed a strong increase in expression for a variety of TE families upon knockdown of *nxf2* (S6 Fig). The 3 telomeric elements, *HeT-A*, *TAHRE*, and *TART-A*, are among the top 10 most highly up-regulated TEs, with *HeT-A* showing approximately 300-fold increase in expression in the *nxf2* knockdown (*TAHRE*: approximately 110-fold increase, *TART-A*: approximately 30-fold increase) (Fig 3). We repeated the experiment using a shRNA that targeted a different region of *nxf2* and observed a similar pattern and strong correlation between TE expression profiles of both knockdowns (Spearman’s rho = 0.94, S7 Fig). The *nxf* paralogs (i.e., *nxf1-4*) are highly diverged (<25% amino acid identity); therefore, it is unlikely that there would be RNAi off-target effects among paralogs. These results support previous findings that *nxf2* is a component of the germline piRNA pathway and show that this gene is particularly important for the suppression of the telomeric TEs HeT-A, TAHRE, and TART-A.

### *TART-A* piRNAs may target *nxf2* for silencing

**Fig 4:**
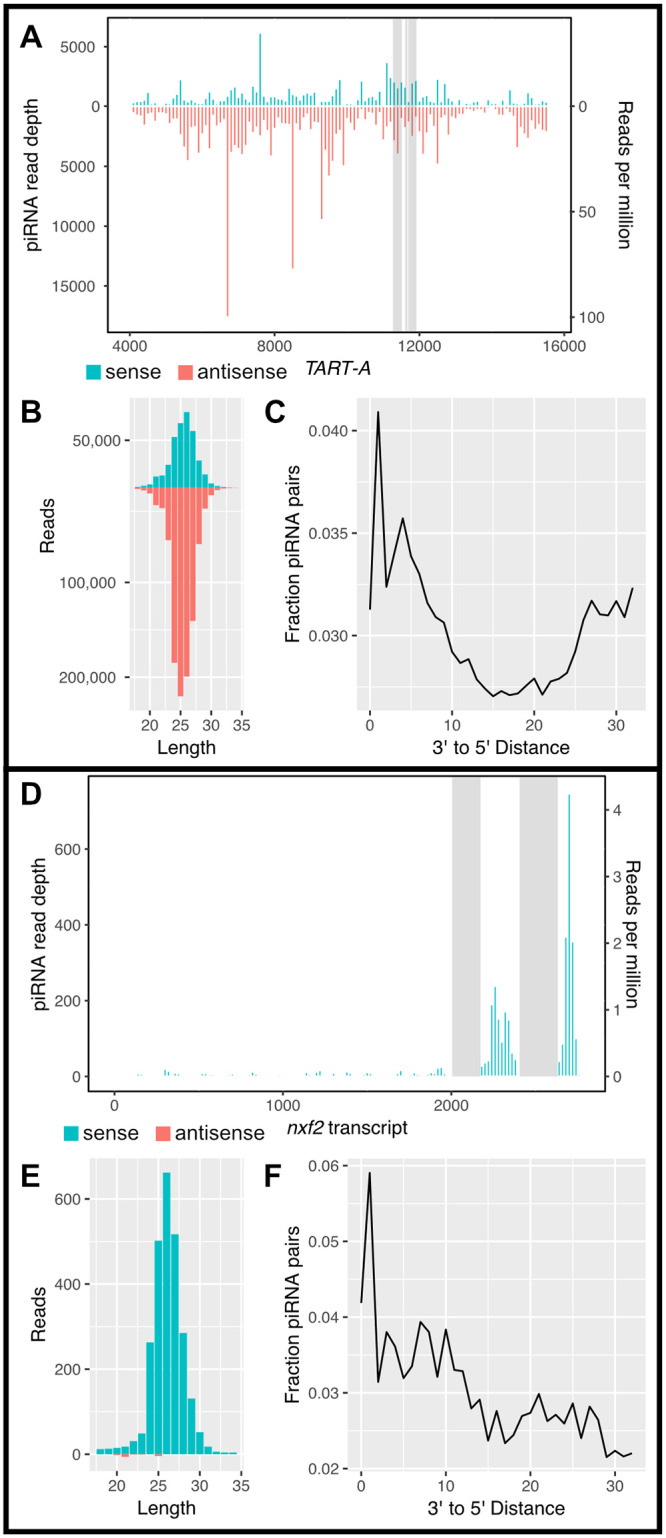
piRNAs are produced from both *TART-A* and *nxf2*. (A) We aligned previously published piRNA data from the *D*. *melanogaster* DGRP [85] to *TART-A* and examined read coverage across the element. We find abundant sense (blue bars) and antisense (red bars) piRNA production across most of the element, including the regions containing the *nxf2*-like sequence (gray boxes). Note that the 5′ UTR of *TART-A* is copied from the 3′ UTR during replication and is therefore identical in sequence. We masked the 5′ UTR (positions 1–4,000) for this analysis. (B) The length of aligned reads are consistent with that expected for piRNAs and the *TART-A* derived piRNAs are biased toward the minus strand. (C) *TART-A* piRNAs show an enrichment of alignments where the 5′ end of 1 piRNA is found directly after the 3′ end of the previous piRNA (i.e., distance of 1), consistent with piRNA phasing. (D) Unlike *TART-A*, *nxf2* produces piRNAs primarily in the regions directly downstream from its *TART*-like sequence (gray boxes). The vast majority of these piRNAs are only from the sense strand of *nxf2* (E) and also show the signature of phasing (F). Note that the *TART*-like sequence of *nxf2* was masked for this analysis to avoid cross-mapping of *TART*-derived piRNAs to the *nxf2* transcript. Underlying data can be found in S2 Data. piRNA, Piwi-interacting small RNA; TE, transposable element.

Previous studies have reported abundant piRNAs derived from the telomeric TEs, *HeT-A*, *TAHRE*, and *TART-A* [62,65,84]. We sought to determine whether piRNAs arising from the *nxf2*-like region of *TART-A* could be targeting the *nxf2* gene for down-regulation via the piRNA pathway. We used previously published piRNA data from 16 wild-derived strains from the DGRP [85]. Among the 16 strains, we found wide variation in *TART-A* piRNA production ranging from 60 to 12,300 reads per million (RPM). From the pool of 16 strains, we identified approximately 1.3 million reads that aligned to *TART-A*, 98% of which map uniquely (see Methods) (Fig 4A). *TART-A* piRNAs have previously been shown to exhibit the 10-bp overlap signature of ping-pong cycle amplification [86], and we identified both sense and antisense piRNAs arising from *TART-A* (Fig 4B) as well as an enrichment of alignments where the 5′ end of 1 piRNA is found directly after the 3′ end of the previous piRNA (i.e., 3′ to 5′ distance of 1), consistent with piRNA phasing (Fig 4C). We identified approximately 95,000 piRNAs arising from the *TART-A* region that shares homology with *nxf2*. Of these reads, 59% are antisense to *TART-A*, and 41% are sense.

We used an allele-specific approach to confirm that these antisense piRNAs are derived from *TART-A* rather than *nxf2*. We identified 17 positions within the region of shared homology where *TART-A* and *nxf2* have different variants. A total of 9,301 bases from *TART*-derived antisense piRNAs aligned to one of these positions, 9,244 (99.4%) of which matched the *TART-A* variant and 27 (0.29%) of which matched the *nxf2* variant. Another 30 bases were different from either *TART-A* or *nxf2* (S1 Table).

We next focused on piRNA production from *nxf2*. We reasoned that, if *nxf2* expression is subject to piRNA-mediated regulation, we should see piRNAs derived from the *nxf2* transcript, outside of the region that shares homology with *TART-A*. We masked the *nxf2*/*TART-A* region of shared homology and aligned the piRNA sequence data to the *nxf2* transcript. We found low but consistent production of piRNAs from *nxf2* across all 16 DGRP strains (between 1.5 and 41 RPM), with 99.7% of *nxf2*-aligned reads mapping uniquely. To increase sequencing depth, we pooled the data from all 16 strains (2,624 *nxf2* reads total) and examined piRNA abundance along the *nxf2* transcript (Fig 4D). We found that the most abundant production of piRNAs from *nxf2* occurs at the 3′ end of the transcript, downstream from the region of shared homology with *TART-A* (Fig 4D). Overall, 99.4% of reads from *nxf2* are derived from the sense strand of the transcript (Fig 4E), and the *nxf2* piRNAs also show evidence of phasing (Fig 4F). We quantified sense-strand piRNA production from all *D*. *melanogaster* protein-coding genes and found that *nxf2* falls within the top 5% of genes in terms of the abundance of sense-strand piRNAs. The enrichment of *nxf2*-derived piRNAs downstream from the region of shared homology with *TART-A*, along with our observation that almost all *nxf2* piRNAs are derived from the sense strand, suggests that these piRNAs are not amplified via the ping-pong cycle, but are instead produced by the Zucchini-mediated phasing process. Furthermore, the lack of antisense piRNAs suggests that *nxf2* is not converted into a dual-strand piRNA cluster [87].

**Fig 5:**
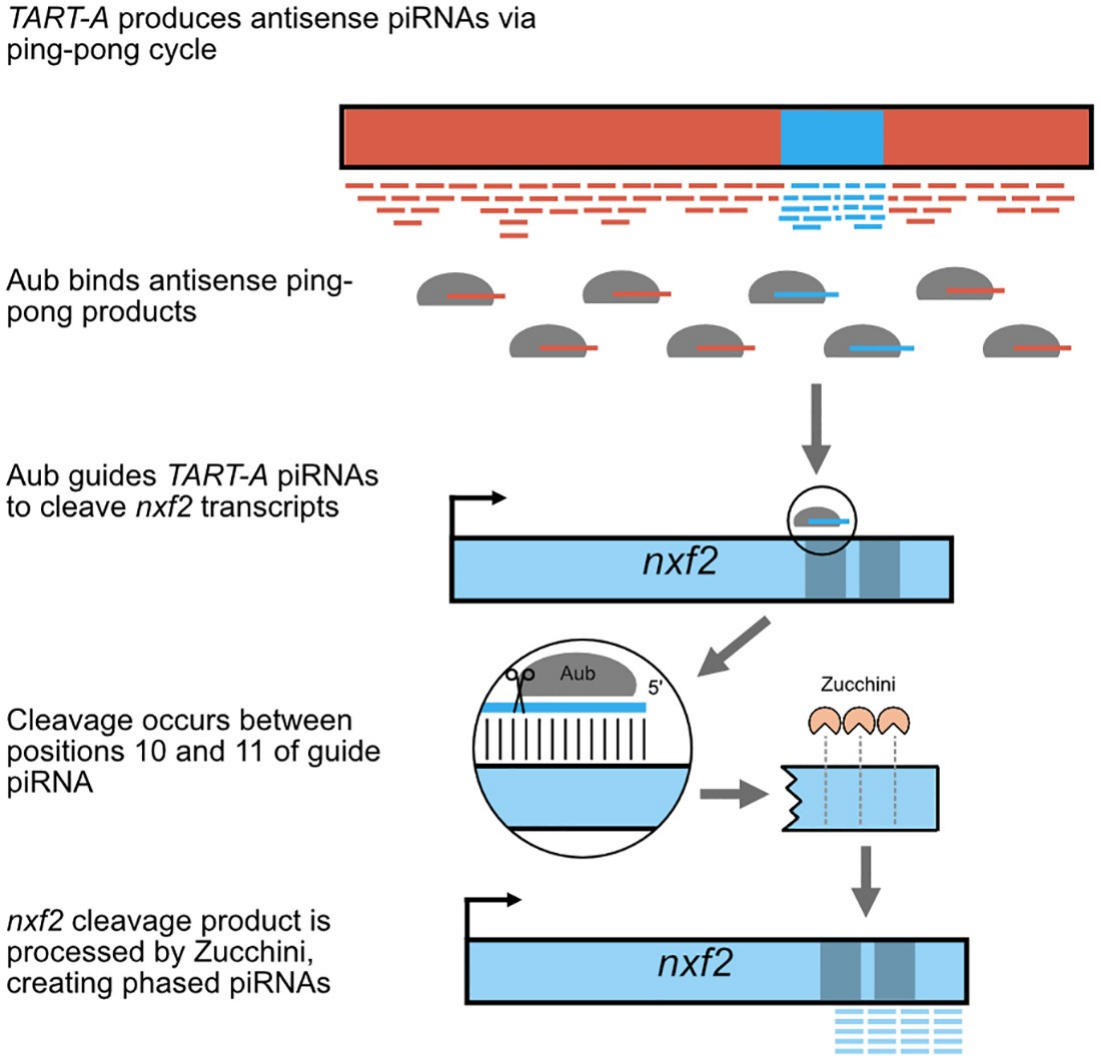
Model describing generation of phased piRNAs from *nxf2*. *TART-A* produces abundant antisense piRNAs derived from ping-pong amplification, including from the *TART-A*/*nxf2* region of shared homology (blue box on red background). The PIWI protein Aubergine binds antisense ping-pong piRNAs, a subset of which share homology with *nxf2*. These piRNAs guide Aub to *nxf2* and result in cleavage of the transcript between the 10th and 11th nucleotide of the guide piRNA. Transcript cleavage creates an *nxf2* cleavage product that shares a 10-bp sense:antisense overlap with the guide piRNA (see S8 Fig). The *nxf2* cleavage product can by subsequently processed by the Zucchini endonuclease, creating phased piRNAs starting from the site of Aub cleavage and proceeding to the 3′ end of the *nxf2* transcript. piRNA, Piwi-interacting small RNA.

These results are consistent with a model where antisense piRNAs from the *nxf2*-like region of *TART-A* are bound by Aubergine and targeted to sense transcripts from the *nxf2* gene. Aub cleaves target transcripts between the bases paired to the 10th and 11th nucleotides of its guide piRNA, resulting in a cleavage product with a 5′ monophosphate that shares a 10-bp sense:antisense overlap with the guide piRNA that triggered the cleavage. These cleavage products can be enriched and sequenced using an approach known as degradome-seq [88]. We analyzed published degradome-seq and Aub-immunoprecipitated piRNA data from wild-type *D*. *melanogaster* ovaries [89] to determine whether we could detect *nxf2* cleavage products resulting from targeting by antisense *TART-A* piRNAs. We first aligned the antisense *TART-A* piRNAs to the *nxf2* transcript, which resulted in 3,601 aligned reads (676 alignments with 0 mismatches, 2,145 with 1 mismatch, and 780 with 2 mismatches). We found 11 locations within the *TART*-like region of *nxf2* where we observe degradome cleavage products that share the characteristic 10-bp sense:antisense overlap with *TART-A* antisense piRNAs (S8 Fig). We performed a permutation test to assess the statistical significance of these overlaps by shuffling the piRNA and degradome-seq read alignments. We found that the degradome-seq alignment locations are associated with more abundant piRNA alignments than expected by chance (*P* = 0.002), and overall, we observe more 10-bp sense:antisense overlaps than expected by chance (*P* = 0.001). These results can be explained under the following model: *TART-A* antisense piRNAs are produced by the ping-pong cycle and bound to Aubergine. A subset of these piRNAs (those from the *nxf2*-like region of *TART-A*) guide Aub to *nxf2* transcripts which are then cleaved. Aub cleavage products can be further processed by Zucchini in the 5′ to 3′ direction, thereby producing phased piRNAs from *nxf2* transcripts downstream from the *nxf2*/*TART-A* regions of shared homology (Fig 5).

**Fig 6:**
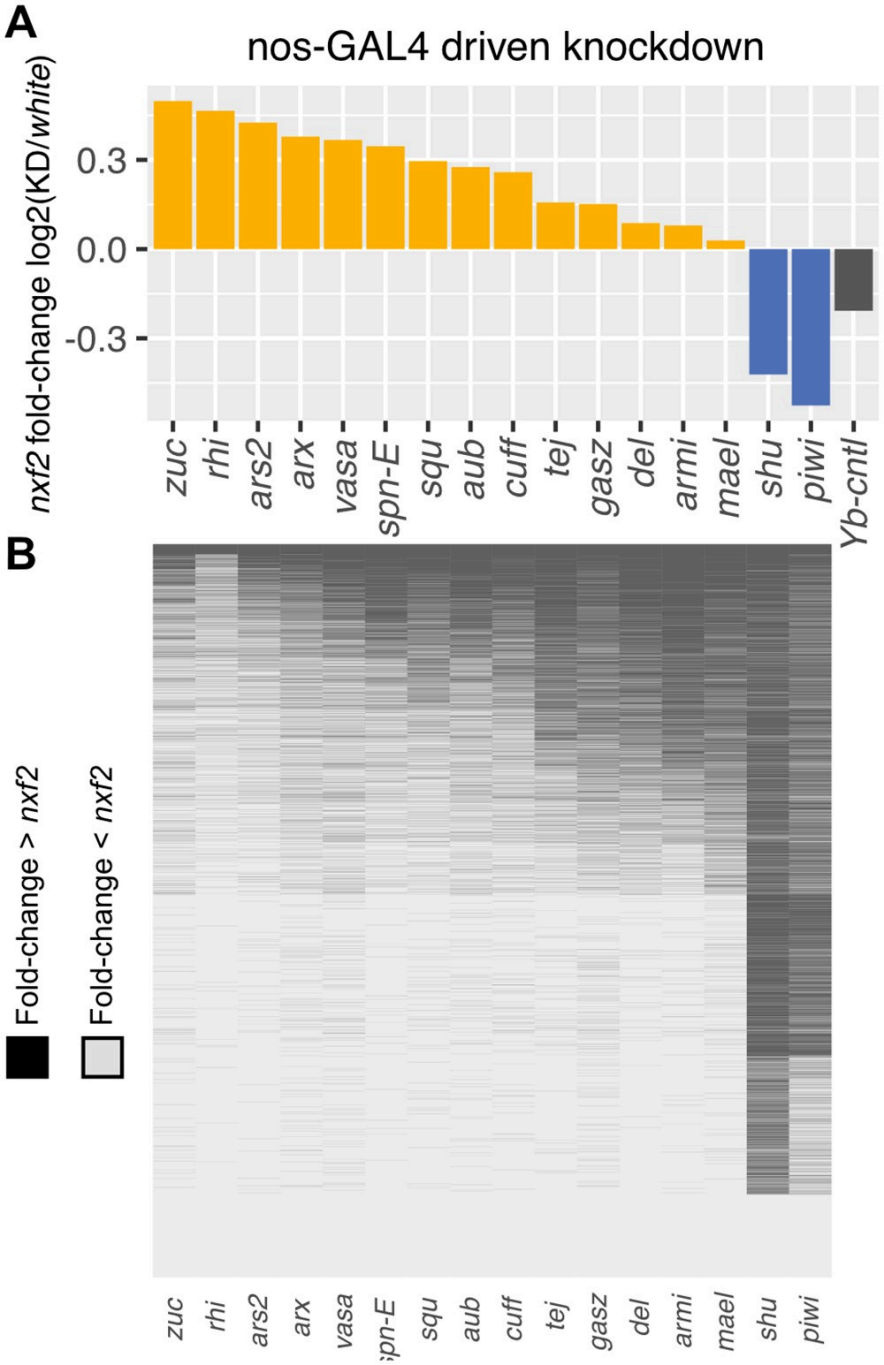
Knockdown of piRNA pathway components is associated with up-regulation of *nxf2*. If *TART*-derived piRNAs are targeting *nxf2* for suppression, disruption of the piRNA pathway should relieve this suppression. We examined previously published RNA-seq data from 16 piRNA component knockdowns, as well as a control (*Yb*) [11]. (A) *Nxf2* expression increased in the majority of the 16 knockdowns (B) We compared the fold change in *nxf2* expression across the 16 knockdowns to all expressed genes. Each row in the heatmap corresponds to a gene and each cell is colored based on whether the fold change in expression for that gene is larger (black) or smaller (gray) than what we observed for *nxf2*. Only 168 genes show larger fold change in expression across all 16 knockdowns, which places *nxf2* in the top 1.4% of expressed genes in terms of its pattern of up-regulation. Underlying data can be found in S2 Data. piRNA, Piwi-interacting small RNA; RNA-seq, RNA sequencing.

If piRNAs from *TART-A* are targeting *nxf2* and down-regulating its expression, knockdown of piRNA pathway components that either decrease piRNA production from *TART-A* (ping-pong and/or phased piRNA pathway components) or disrupt silencing of *nxf2* (phased piRNA components) should result in an increase in expression of *nxf2*. We analyzed published RNA sequencing (RNA-seq) data from nos-GAL4 driven knockdowns of 16 genes that were identified as components of the piRNA pathway and that were specifically shown to be involved in repression of *HeT-A* and *TAHRE* [11]. We compared the expression of *nxf2* in each piRNA component knockdown to its expression in the control knockdown of the *white* gene and found that *nxf2* shows increased expression in the majority of knockdowns; however, the observed increase in *nxf2* expression is relatively mild (Fig 6A).

The small change in expression is likely due to the fact that the *nxf2* expression data are from bulk ovaries containing both somatic and germ cells, whereas the knockdown is germline specific. *Nxf2* is expressed at much higher levels in somatic follicle cells compared to germ cells [6]. The somatic expression, which should be unchanged between knockdown and control, will therefore mask a larger fold change that is specific to the germline. However, *nxf2* should still show an increased fold change in expression relative to other genes in the genome, even though the true magnitude of expression fold change may be obscured by the mix of somatic and germ cells. We therefore compared the fold change in *nxf2* expression to the genome-wide pattern of fold changes to assess the significance of *nxf2* up-regulation.

For each gene with nonzero expression in the control knockdown, we determined whether its fold change in expression was greater than or equal to that of *nxf2*, for each of the 16 piRNA component knockdowns. We found only 168 genes with fold changes equal to or larger than *nxf2* for each of the 16 knockdowns, which places *nxf2* among the top approximately 1.4% of expressed genes in terms of its pattern of up-regulation (Fig 6B). Interestingly, in wild-type DGRP strains, these 168 genes have significantly more piRNAs aligning to them, compared to the remainder of expressed genes, suggesting their expression may also be regulated by piRNAs (Wilcoxon test *P* = 4.1e-06) (S9 Fig).

For the same 16 knockdown experiments, we also assessed the pattern of *nxf2* up-regulation relative to other known piRNA pathway genes. We obtained a list of 41 germline-specific and germline/soma piRNA pathway genes from [90]. None of these 41 genes are among the 168 genes with larger fold changes than *nxf2*. To further confirm that there is not a general up-regulation of piRNA pathway genes upon disruption of the piRNA pathway, we examined the expression of these 41 genes for each of the 16 piRNA component knockdowns, excluding the targeted gene from analysis for each knockdown. We found no evidence of a uniform up-regulation of piRNA pathway genes across each knockdown. Instead, the median fold change of piRNA pathway genes is near 1 for each experiment (S10 Fig).

### Natural variation in *TART-A* copy number is correlated with *nxf2* expression levels

**Fig 7:**
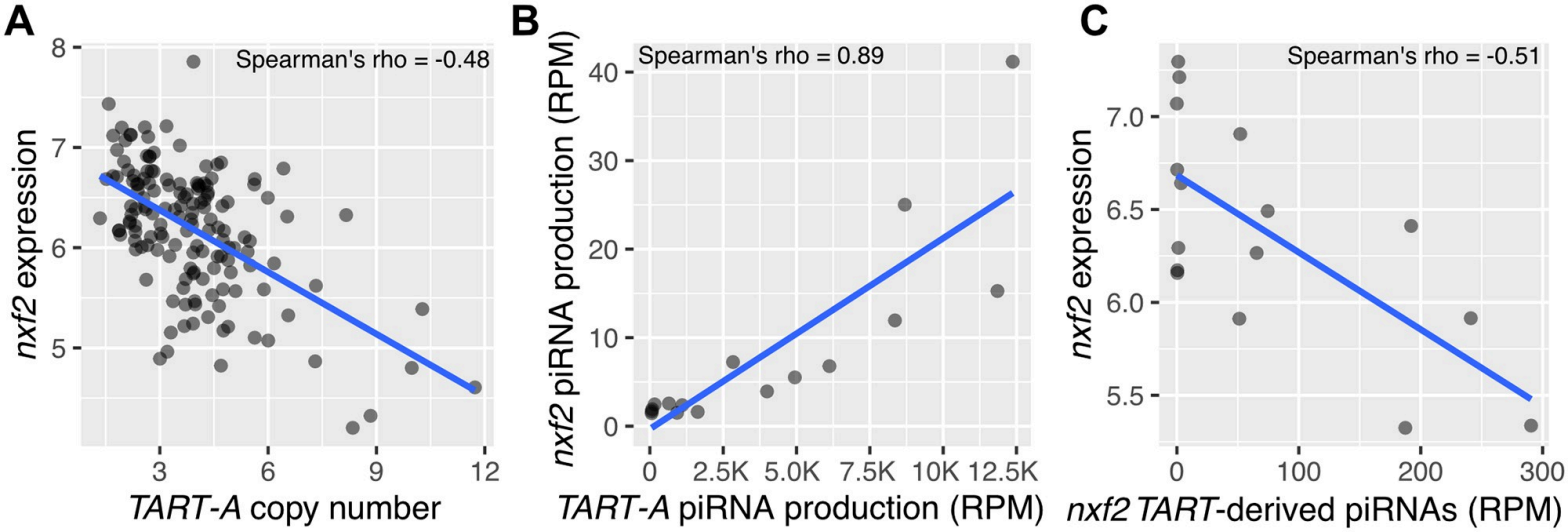
*TART-A* copy number is negatively correlated with *nxf2* expression across the DGRP. (A) We inferred *TART-A* copy number for 151 DGRP strains using published Illumina sequencing data [80,81] and retrieved expression values for *nxf2* from microarray data from whole adult females [125]. We found that *TART-A* copy number is significantly negatively correlated with *nxf2* expression levels, as expected if *TART-A* piRNAs are targeting *nxf2* for suppression (Spearman’s rho = −0.48, *P* = 4.6e-10). (B) We also compared piRNA production from *TART-A* and *nxf2* across 16 DGRP individuals using data from [85]. There is a strong positive correlation between *TART-A* piRNA production and *nxf2* piRNA production (Spearman’s rho = 0.89, *P* < 2.2e-16) across the 16 DGRP strains. (C) We also compared *nxf2* expression to the amount of *TART*-derived piRNAs that align to *nxf2* across these same strains. In this case, we observe a negative correlation, slightly larger in magnitude than what we see for our comparison of *nxf2* expression and *TART-A* copy number (Spearman’s rho = −0.51, *P* = 0.046). Underlying data can be found in S2 Data. DGRP, *Drosophila* Genetic Reference Panel; piRNA, Piwi-interacting small RNA; RPM, reads per million.

Previous work has shown that there is a large variation in HTT element copy number at the telomeres of wild *Drosophila* strains [68,91]. Our results predict that, if *TART-A* piRNAs are targeting *nxf2* for suppression, then strains with more copies of *TART-A* should have lower expression of *nxf2* and vice versa: Isofemale lines with low *nxf2* expression should accumulate more copies of *TART-A*. To test this prediction, we used previously published Illumina genomic sequencing data and microarray gene expression profiles from the DGRP [80,81]. We used the Illumina data to infer *TART-A* copy number for 151 DGRP strains (see Methods) and obtained *nxf2* microarray gene expression levels from whole adult females for these same strains. *Nxf2* is predominantly expressed in the ovary [6]; therefore, the expression of *nxf2* in whole females mainly reflects the expression of *nxf2* in the ovary. We found that, as predicted, there is a strong negative correlation between *TART-A* copy number and *nxf2* gene expression levels among the DGRP (Fig 7A) (Spearman’s rho = −0.48, *P* = 4.6e-10). We obtained a similar result using a replicate microarray dataset (S11 Fig). Furthermore, this pattern is unique to *nxf2*: correlation coefficients comparing *TART-A* copy number to gene expression of other piRNA pathway components are at least 2-fold smaller in magnitude (S12 and S13 Figs).

We predict that *TART-A* piRNAs are targeting *nxf2* for suppression, leading to the production of additional *nxf2*-derived piRNAs downstream from the region of shared homology. Therefore, DGRP strains with larger production of *TART-A* piRNAs should also have larger amounts of *nxf2*-derived piRNAs. Consistent with this expectation, we find a strong positive correlation between *TART-A* piRNA production and *nxf2* piRNA production (Spearman’s rho = 0.89, *P* < 2.2e-16) across 16 DGRP strains [85] (Fig 7B, S14 Fig). We find a similar correlation when we compare the *TART*-derived piRNAs that align to *nxf2* versus the *nxf2* piRNAs downstream from the region of shared homology (Spearman’s rho = 0.88, *P* < 2.2e-16) (S15 Fig) We also compared *nxf2* expression to the amount of *TART*-derived piRNAs that align to *nxf2* across these same strains. In this case, we observe a negative correlation, slightly larger in magnitude than what we see for our comparison of *nxf2* expression and *TART-A* copy number (Spearman’s rho = −0.51, *P* = 0.046) (Fig 7C).

### Evidence for genetic conflict between *nxf2* and *TART-A*

**Fig 8:**
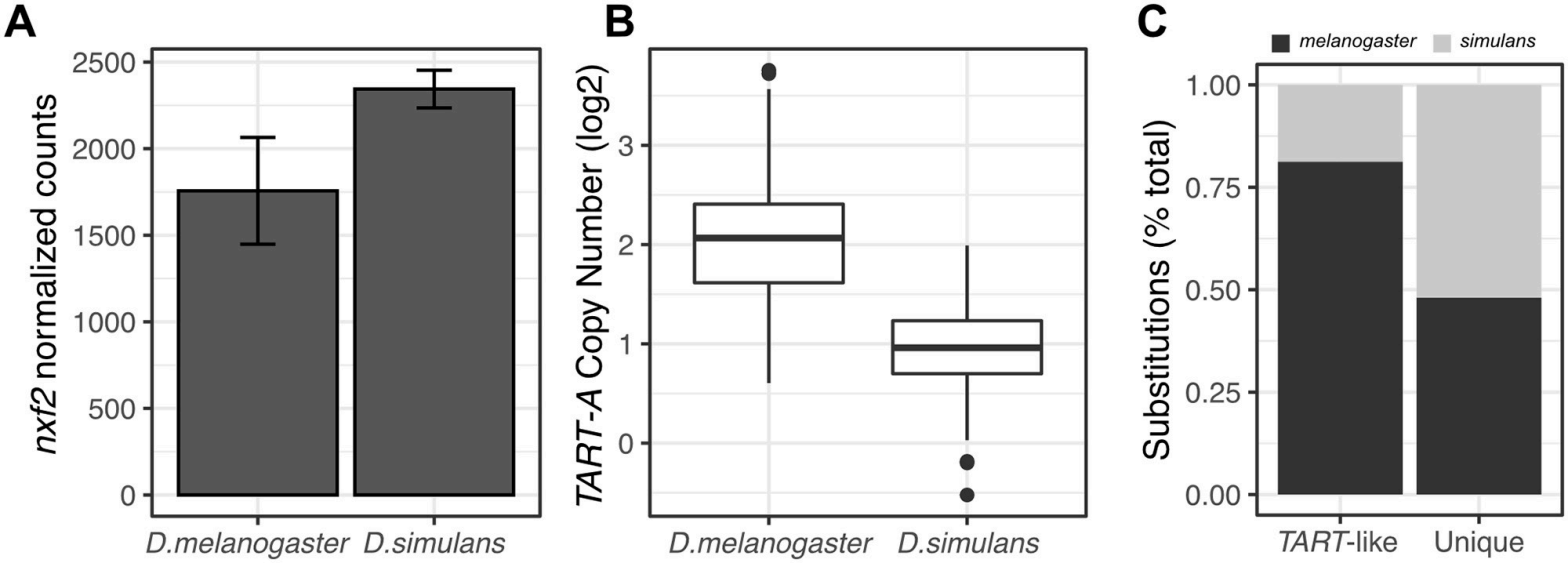
Evidence for genetic conflict between *nxf2* and *TART-A*. (A) We used mRNA-seq from ovaries to compare *nxf2* expression between *D*. *melanogaster* and *D*. *simulans* (5 isofemale lines per species). We found that *nxf2* expression in *D*. *melanogaster* is significantly reduced compared to *D*. *simulans*, consistent with *D*. *melanogaster*–specific repression (Wilcoxon test *P* = 0.031). Bars show mean expression level and whiskers show standard deviation. (B) We inferred *TART-A* copy number using genomic sequencing data from 90 *D*. *simulans* individuals [93]. We found that the *D*. *melanogaster* DGRP population has a significantly larger number of *TART-A* copies per individual (1–13 copies per strain, median = 4) compared to the *D*. *simulans* population (1–4 copies per strain, median = 2) (Wilcoxon test *P* < 2.2e-16). (C) We identified lineage-specific mutations in *nxf2* CDS for both *D*. *melanogaster* and *D*. *simulans* using *D*. *yakuba* as an outgroup and used Tajima relative rate test to compare the evolutionary rate of *nxf2* since it diverged from the common ancestor of *D*. *melanogaster* and *D*. *simulans*. For the *TART*-like region of *nxf2*, the evolutionary rate is significantly accelerated along the *D*. *melanogaster* branch, relative to *D*. *simulans* (13 *D*. *melanogaster*–specific substitutions, 3 *D*. *simulans* substitutions, *P* = 0.012), whereas there is no difference in evolutionary rate for the remainder of the gene (37 *D*. *melanogaster*–specific substitutions, 40 *D*. *simulans*–specific substitutions, *P* = 0.73). Underlying data can be found in S2 Data. CDS, coding sequence; mRNA-seq, mRNA sequencing.

If *nxf2* is being repressed by *TART-A* specifically in *D*. *melanogaster*, *nxf2* expression should be reduced in *D*. *melanogaster* relative to other closely related species. We performed mRNA sequencing (mRNA-seq) of ovaries for *D*. *simulans* strain *w501* as well as 5 DGRP strains whose median *nxf2* expression level is similar to that of the population as a whole based on the DGRP microarray data (S16 Fig). *Nxf2* showed reduced expression in all 5 DGRP strains relative to *D*. *simulans* (average *melanogaster*/*simulans* fold change: 0.76). We then reanalyzed previously published mRNA-seq data from the ovaries of 4 other *D*. *simulans* strains [92], corrected for batch effects, and compared *nxf2* expression between the 2 species. We found that *nxf2* expression in *D*. *melanogaster* is significantly reduced compared to *D*. *simulans*, consistent with *D*. *melanogaster*–specific repression (*P* = 0.0039) (Fig 8A). If reduced *nxf2* expression in *D*. *melanogaster* relative to *D*. *simulans* increases *TART-A* activity, then *TART-A* should show higher copy numbers in *D*. *melanogaster*. We inferred *TART-A* copy number using genomic sequencing data from 90 individuals from a North American population of *D*. *simulans* [93]. We found that the *D*. *melanogaster* DGRP population has a significantly larger number of *TART-A* copies per individual (1 to 13 copies per strain, median = 4) compared to the *D*. *simulans* population (1 to 4 copies per strain, median = 2) (Wilcoxon test *P* < 2.2e-16) (Fig 8B).

If *nxf2* is expressed at suboptimal levels due to repression by *TART*-derived piRNAs, selection should favor mutations within the *TART*-like region of *nxf2* that reduce the amount of shared homology. To test for this pattern, we identified lineage-specific mutations in *nxf2* CDS for both *D*. *melanogaster* and *D*. *simulans* using *D*. *yakuba* as an outgroup. If *nxf2* is evolving to escape *TART*-mediated suppression, we would expect an accelerated rate of evolution of *D*. *melanogaster nxf2* specifically within the region of shared homology, since its common ancestor with *D*. *simulans*. We used the Tajima relative rate test [94] to compare the evolutionary rate of *nxf2* since it diverged from the common ancestor of *D*. *melanogaster* and *D*. *simulans*. For the *TART*-like region of *nxf2*, we found that the evolutionary rate is significantly accelerated along the *D*. *melanogaster* branch, relative to *D*. *simulans* (13 *D*. *melanogaster*–specific substitutions, 3 *D*. *simulans* substitutions, χ^2^ test statistic = 6.25, *P* = 0.012, 1 degree of freedom), whereas there is no difference in evolutionary rate for the remainder of the gene (37 *D*. *melanogaster*–specific substitutions, 40 *D*. *simulans*–specific substitutions, χ^2^ test statistic = 0.12, *P* = 0.73, 1 degree of freedom) (Fig 8C). We also found that the majority of the *D*. *melanogaster*–specific substitutions in the *TART*-like region of *nxf2* occurred after the capture of *nxf2* by *TART-A*: Of the 13 *D*. *melanogaster*–specific substitutions, *TART-A* is identical to *D*. *simulans* at 10 of the sites, and identical to *D*. *melanogaster* at only 3 sites (S1 Data), consistent with *D*. *melanogaster nxf2* evolving away from *TART-A*.

## Discussion

If the CDS of a gene shares sequence homology with a known TE, the most likely explanation for this shared homology is that a portion of the gene was derived from a TE insertion. This is, understandably, what was previously reported by Sackton and colleagues for the *nxf2* gene and the *TART-A* TE [71]; however, our analyses are not consistent with such a scenario. Specifically, based on sequence similarity and phylogenetic clustering, the event that created the shared homology between *nxf2* and *TART-A* must have occurred relatively recently, after *D*. *melanogaster* diverged from *D*. *simulans*, yet the putative insertion of *TART-A* in the *nxf2* gene is shared across *Drosophila*.

A scenario that is more consistent with these observations is one where, rather than the *nxf2* gene gaining sequence from *TART-A*, the *TART-A* element captured a portion of the *nxf2* gene, likely via aberrant transcription that extended past the internal *TART-A* poly-A signal to another poly-A signal in the flanking genomic region. This process has been observed for other TEs and is known as exon shuffling or transduction [76,77]. Notably, the *nxf2*-like sequence of *TART-A* is located in its 3′ UTR, which would be expected if it were acquired via transduction (Fig 1). Interestingly, *TART* is part of the LINE family of non-LTR retrotransposons, and Human LINE-L1 elements are known to undergo transduction fairly frequently [76,77,95]. However, transduction would require that an active *TART-A* element was inserted somewhere upstream of the 3′ region of *nxf2* at some point in the *D*. *melanogaster* lineage but has since been lost from the population. Is this possible given that *TART-A* should only replicate to chromosome ends? The TIDAL-fly database of polymorphic TEs in *D*. *melanogaster* reports several polymorphic *TART-A* insertions far from the chromosome ends, which suggests that this element is occasionally capable of inserting into locations outside of the telomeres [96]. The aberrant *TART-A* copy that acquired a portion of the *nxf2* gene likely arose as a single polymorphic insertion in an ancestral *D*. *melanogaster* population yet has now probably replaced the ancestral *TART-A* element: Our results show that the *nxf2*-like region of *TART-A* is now present in most, if not all, full-length *TART-A* elements in *D*. *melanogaster*.

*Nxf2* suppresses *TART-A* activity via its role in the piRNA pathway, whereas the acquisition of *nxf2* sequence appears to allow *TART-A* to suppress *nxf2*. Our results are consistent with a scenario where TART-derived piRNAs guide Aub proteins to the *nxf2* transcript. The *TART-A* piRNAs may then act as “trigger” piRNAs that catalyze cleavage of *nxf2* transcripts while also resulting in the production of phased piRNAs starting in the region of shared homology and proceeding in the 3′ direction to the end of the *nxf2* transcript (Fig 5). The piRNA-mediated cleavage of *nxf2* transcripts, which is supported by degradome-seq data (see S8 Fig), should result in a reduction in *nxf2* expression levels.

The fact that Nxf2 is known to interact with Piwi-targeted transcripts makes it very likely that it is directly interacting with *TART-A*. Telomeric piRNAs are bound by Piwi [63,97,98], and Piwi has previously been described as playing an important role in maintenance of telomeric chromatin: In *piwi* mutants, the telomeres become depleted of H3K9me3 and move from the nuclear periphery to the interior [99]. Interestingly, Zhao and colleagues observed a similar translocation of telomeres to the nuclear interior in their *nxf2* mutant [9].

Given that *nxf2* plays a role in suppressing *TART-A* activity, reduced *nxf2* levels should relieve *TART-A* suppression, which would presumably increase *TART-A* fitness by allowing it to make more copies of itself. Indeed, in the DGRP, we find that individuals with lower *nxf2* expression levels tend to have higher numbers of *TART-A* copies and vice versa (Fig 7). If additional copies of *TART-A* act to further suppress *nxf2* expression, which then further derepresses *TART-A*, why is there not run-away accumulation of telomere length in *D*. *melanogaster*? Previous work has shown that long telomeres in *D*. *melanogaster* are associated with both reduced fertility and fecundity [91], so it is possible that a run-away trend toward increasing telomere length is balanced by a fitness cost.

Our results provide several lines of evidence that *nxf2* and *TART-A* are evolving in conflict. In *D*. *melanogaster*, *nxf2* is expressed at lower levels and *TART-A* has proliferated to higher copy numbers compared to *D*. *simulans*, consistent with *TART-A* benefitting from *nxf2* suppression (Fig 8B and 8C). If *nxf2* expression level is suboptimal in *D*. *melanogaster* due to *TART-A* suppression, there should be selection to disrupt the shared homology between these 2 elements, which is supported by our finding that the *TART*-like region of *nxf2* is experiencing accelerated evolution in the *D*. *melanogaster* lineage (Fig 8A).

Targeting of host transcripts by transposon-derived piRNAs has been previously observed in *Drosophila*. Most notably, piRNAs from the LTR retrotransposons *roo* and *412* play a critical role in embryonic development by targeting complementary sequence in the 3′ UTR of the gene *nos*, leading to its repression in the soma [100]. More recent results suggest hundreds of maternal transcripts could be regulated in a similar fashion [101]. However, these represent cases where TE piRNAs have been co-opted to regulate host transcripts, whereas our results suggest that the piRNA targeting of *nxf2* is a counter-defense strategy by *TART-A*. This type of strategy has only been previously observed in plants [32]. In rice, a CACTA DNA transposon produces a micro-RNA that targets a host methyltransferase gene known to be involved in TE suppression [28], while in Arabidopsis, siRNAs from *Athila6* retrotransposons target the stress granule protein UBP1b, which is involved in suppressing *Athila6* GAG protein production [29].

Given that viruses and other pathogens have evolved a variety of methods to block or disrupt host defense mechanisms, it is surprising that there is much less evidence for TEs adopting similar strategies [32]. However, unlike viruses, TEs depend heavily on vertical transmission from parent to offspring. Any counter-defense strategy that impacts host fitness would therefore decrease the fitness of the TE as well. Our finding that the *nxf2*-like region of *TART-A* appears to be fixed in *D*. *melanogaster* is therefore unexpected: *TART-A* variants lacking *nxf2* homology should benefit from the presence of the *nxf2*-like *TART-A* element without incurring the fitness cost. The *nxf2*-like *TART-A* variant should therefore be under frequency dependent selection and would be expected to remain polymorphic. One possibility that would explain the fixation of the novel *TART-A* variant is if the *nxf2*-like sequence also enhances *TART-A* retrotransposition. Unusually long UTRs are a hallmark of all *HTT* elements that have been identified across multiple *Drosophila* species; however, their specific functional role in retrotransposition remains unknown.

Another reason that TE counter-defense may be rare is that disruption of host silencing is likely to lead to up-regulation of other TEs, making it more likely that there will be a severe decrease in host fitness, similar to what is observed in hybrid dysgenesis. *TART-A* may be targeting *nxf2* for its own advantage, but our knockdown experiment shows that *nxf2* suppression causes up-regulation of many other TEs besides *TART-A* (Fig 3, S6 Fig), and other studies have shown that *nxf2* mutants are sterile [6, 7]. Why then, does *TART-A* appear to be targeting *nxf2* in spite of these potentially deleterious consequences? One possibility is that the suppression of *nxf2* expression caused by *TART-A* is relatively mild (i.e., much less than the level of down-regulation caused by the RNAi knockdown), which is enough to provide a slight benefit to *TART-A* without causing widespread TE activation. It is also possible that the suppression effect was initially much larger but has since been counterbalanced by *cis*-acting variants that increase *nxf2* expression and/or reduction in shared homology caused by substitutions in the *TART*-like region of *nxf2*. Future work examining TE activation under varying levels of *nxf2* expression may help to determine whether there is a tipping point where *nxf2* suppression becomes catastrophic.

In summary, our results show that so-called domesticated TEs, if active, can still be in conflict with their host and raise the possibility that TE counter-defense strategies may be more common than previously recognized, despite the potentially deleterious consequences for the host.

## Methods

### *TART-A* sequence analysis

We used the *TART-A* sequence from RepBase [102], which is derived from the sequence reported in [73] (GenBank accession AJ566116). This sequence represents a single full-length *TART-A* element cloned from the *D*. *melanogaster* iso1 reference strain. The *nxf2*-like portion of this sequence is 100% identical to another *TART-A* element cloned and sequenced from *D*. *melanogaster* strain A4-4 (GenBank DMU02279) [53] as well as the *TART-A* sequence from the FlyBase canonical set of transposon sequences (version 9.42) [103] (cloned from *D*. *melanogaster* strain Oregon-R: GenBank AY561850) [104].

We used BLAST [72] to compare the *TART-A* sequence to the *D*. *melanogaster nxf2* transcript and visualized BLAST alignments with Kablammo [105]. To compare *TART-A* among *Drosophila* species, we used the *D*. *yakuba TART-A* sequence reported in [69](GenBank AF468026), which includes the 3′ UTR. We also used the *D*. *sechellia TART-A* ORF2 reported by [59] (GenBank AM040251) to search the *D*. *sechellia* FlyBase r1.3 genome assembly for a *TART-A* copy that included the 3′ UTR, which we found on scaffold_330:4944–14419. We attempted a similar approach for *D*. *simulans*, but were unable to find a *TART-A* copy in the *D*. *simulans* FlyBase r2.02 assembly that included the 3′ UTR. We aligned the *D*. *melanogaster*, *D*. *yakuba*, and *D*. *sechellia TART-A* sequences to each other, and to the *D*. *melanogaster nxf2* transcript (FlyBase FBtr0089479), using nucmer [106]. We then used mummerplot [106] to create a dotplot to visualize the alignments.

To determine whether the *TART-A nxf2*-like sequence was fixed or polymorphic in *D*. *melanogaster*, we used BLAST (e-value cutoff 1e-50) to search the *TART-A* 3′ UTR against genome assemblies from the following *D*. *melanogaster* strains: the iso1 release 6 reference genome [107], nanopore assemblies for DGRP379 and DGRP732 [79], and PacBio (Pacific Biosciences, Menlo Park, California, USA) assemblies for 14 *D*. *melanogaster* strains [78]. We extended the coordinates of each BLAST hit to match the length of the full UTR and extracted the sequences from their assemblies. We then further filtered them by requiring each sequence to have at least 1,000 aligned bases between it and the *TART-A* RepBase sequence, and we required the best BLAST hit of each sequence to be *TART-A*, when searched against the RepBase TE database. We then created a multiple sequence alignment of all sequences using MAFFT [108].

### nxf2 sequence analysis

We downloaded *nxf2* transcripts from the NCBI RefSeq database for *D*. *simulans* (XM_016169386.1), *yakuba* (XM_002095083.2), *erecta* (XM_001973010.3), *biarmipes* (XM_017111057.1), and *elegans* (XM_017273027.1) and created a codon-aware multiple sequence alignment using PRANK [109], which we visualized with JalView [110]. To compare Nxf2 peptide sequences, we used the web version of NCBI BLAST to search the *D*. *melanogaster* Nxf2 peptide sequence against all *Drosophila* peptide sequences present in the RefSeq database. We then used the NCBI COBALT [111] multiple-sequence alignment tool to align the sequences shown in S1 Fig. We used MEGA [112] to conduct Tajima relative rates test for *nxf2*.

### *TART-A*/*nxf2* gene tree

We extracted the *nxf2*-like sequences from all *TART-A* copies present in the *D*. *melanogaster* reference genome and aligned them to the *TART*-like *nxf2* sequences from 7 *Drosophila* species using PRANK. We then inferred a maximum likelihood phylogeny with 100 bootstrap replicates using RAxML [113].

### *nxf2* knockdown

We used 2 different strains from the *Drosophila* TRiP that express dsRNA for RNAi of *nxf2* (Bloomington #34957 and #33985), as well as a control strain for RNAi of the *white* gene (Bloomington #33613). These 3 RNAi strains were all generated from the same *attP2* progenitor strain. Seven males of each of these strains were crossed to seven, 3- to 5-day-old, virgin females carrying the nos-GAL4 driver (Bloomington #25751). After 6 days of mating, we discarded the parental flies and then transferred F1 offspring to fresh food for 2.5 days before collecting ovaries from 6 females for each cross. We performed 2 biological replicates for each of the 3 crosses, dissected the ovaries in 1× PBS and immediately transferred them to RNAlater. We extracted RNA using Trizol/Phenol-Chloroform and used the AATI Fragment Analyzer to assess RNA integrity. We then prepared stranded, total RNA-seq libraries by first depleting rRNA with ribo-zero and then using the NEBnext ULTRA II library prep kit (New England Biolabs, Ipswich, Massachusetts, USA) to prepare the sequencing libraries. The libraries were sequenced on the Illumina NextSeq machine with 150-bp paired-end reads.

### *nxf2* knockdown RNA-seq analysis

The average insert sizes of the total RNA-seq libraries were less than 300 bp, which resulted in overlapping mate pairs for the majority of sequenced fragments. Instead of analyzing these data as paired-end reads, we instead merged the overlapping mates to generate single-end reads using BBmerge [114]. We removed rRNA and tRNA contamination from the merged reads by aligning them to all annotated rRNA and tRNA sequences in the *D*. *melanogaster* reference genome using Hisat2 [115] and retained all unaligned reads. In order to quantify expression from genes as well as TEs, we combined all *D*. *melanogaster* transcript sequences (FlyBase version 6.26) with *D*. *melanogaster* RepBase TE consensus sequences. We accounted for multi-mapping reads by using bowtie2 [116] to align each read to all possible alignment locations (using—*all* and—*very-sensitive-local*) and then using eXpress [117] to estimate FPKM values, accounting for the multi-mapped alignments. We averaged FPKM values between biological replicates and assessed the reproducibility of both TE and gene expression profiles in the *nxf2* knockdown by comparing the results from the 2 different dsRNA hairpins.

### piRNA analysis

We analyzed previously published piRNA data from 16 strains from the DGRP [85]. We used cutadapt [118] to trim adapter sequences from each library and then removed rRNA and tRNA sequences by using bowtie [119] to align the reads to all annotated rDNA and tRNA genes in the *D*. *melanogaster* reference genome, retaining the reads that did not align. We then created a reference database composed of the following sequence sets: a hard-masked version of the *D*. *melanogaster* reference genome assembly (release 6) where all TE sequences and the *nxf2* gene were replaced by N’s using RepeatMasker, the full set of *D*. *melanogaster* RepBase TE consensus sequences, and the *nxf2* transcript, with its *TART*-like region replaced by N’s. Because the 5′ UTR is copied from the 3′ UTR, we also masked the 5′ UTR of *TART-A*. We used the unique-weighting mode in ShortStack [120,121] to align the piRNA reads to this reference database. With this mode, ShortStack probabilistically aligns multi-mapping reads based on the abundance of uniquely mapping reads in the flanking region. We then used the ShortStack alignments and Bedtools [122] to calculate coverage for sense and antisense alignments to *TART-A* as well as *nxf2*. To test for evidence of piRNA phasing, we used the formula described in [123].

### piRNA component knockdowns

We used the raw read counts reported in GEO accession GSE117217 from 16 RNAi knockdowns of piRNA pathway components as well as a control knockdown of the *Yb* gene [11]. We used the DESeq2 median of ratios approach to normalize raw counts [124].

### Degradome-seq analysis

We used degradome-seq and Aub-immunoprecipitated small RNA data from wild-type *D*. *melanogaster* strain *w1* [89]. We used bowtie2 to align the degradome-seq data to the same reference sequence used in the piRNA analysis except we unmasked the *nxf2* transcript. The degradome-seq data are 100-bp paired-end reads which are long enough to distinguish between the *TART*-like region of *nxf2* and the *nxf2*-like region of *TART-A*. We analyzed the small RNA data as described under “piRNA analysis,” except we allowed 2 mismatches for alignments between *TART*-derived piRNAs and *nxf2*. We then used Bedtools to extract degradome read alignments whose 5′ end was located in the *TART*-like region of *nxf2* and antisense small RNA alignments whose 5′ end was located in the *nxf2*-like region of *TART-A* and whose length was consistent with piRNAs (23 to 30 bp). We then used bowtie to align the minus strand piRNAs to the *nxf2* transcript and used bedtools to identify piRNAs whose 5′ end overlapped the 5′ of degradome reads by 10 bp.

For the permutation test, we determined the number of antisense piRNAs whose 5′ ends aligned at each position within the *TART*-like region of *nxf2*. From the degradome-seq alignments, we determined there were 18 unique locations within the *TART*-like region of *nxf2* where at least 1 degradome-seq read aligned. Eleven of these locations also showed the 10-bp sense:antisense overlap with at least 1 piRNA. To test whether the 10-bp sense:antisense overlaps that we observed between degradome and piRNA reads were associated with higher abundance piRNAs, we randomly sampled 11 piRNA alignment locations from the *TART*-like region of *nxf2* and calculated the number of piRNAs that aligned at each location using Bedtools. We repeated this process 1,000 times and counted the number of times where the random sample had the same or more piRNAs at each location compared to the true alignments. To test whether we observed more 10-bp sense:antisense overlaps than expected by chance, we randomly sampled 18 positions within the *TART*-like region of *nxf2* and counted how many of the 18 positions also had a 10-bp sense:antisense overlap with at least 1 piRNA. We repeated this process 1,000 times and determined the number of times where the random sample had the same or more 10-bp sense:antisense overlaps compared to the true sample.

### *TART-A* copy number variation and *nxf2* expression

We obtained *nxf2* expression values from previously published microarray gene expression profiles from whole adult females for all DGRP strains [125] and used Illumina genomic sequencing data from the DGRP [80,81] to estimate *TART-A* copy number. Across strains, the DGRP Illumina data differs in terms of coverage, read length, and paired versus single-end data. To attempt to control for these differences, we trimmed all reads to 75 bp and treated all data as single-end. We also downsampled all libraries to approximately 13 million reads. We first trimmed each strain’s complete dataset (unix command: *zcat file*.*fastq*.*gz | cut -c 75*) and then aligned the trimmed reads to the *D*. *melanogaster* release 6 genome assembly using bowtie2 with the—*very-sensitive* option. We then corrected the resulting bam file for GC bias using DeepTools [126] and counted the number of aligned reads in the corrected bam file using samtools [127]. We removed all strains with less than 13 million aligned reads and, for each remaining strain, we calculated the fraction of reads to keep by dividing the smallest number of aligned reads across all remaining individuals (13,594,737) by the total number of aligned reads for that strain. We then used this fraction to randomly downsample the GC corrected bam file using the *subsample* option from *samtools view* [127]. We converted each bam file to a fastq file with *samtools fastq* and aligned the fastq file to the *D*. *melanogaster* RepBase TE sequences with *bowtie2* using the—*very-sensitive*,—*local*, and—*all* options. With—*all*, *bowtie2* reports every possible alignment for each multi-mapping sequence. We then used *eXpress* to retain a single alignment for each multi-mapping sequence based on the abundance of neighboring unique alignments. We used the *eXpress* bam files to calculate the median per-base coverage (excluding positions with coverage of 0) for the *TART-A* CDS (i.e., ORF1 and ORF2), for each individual. To estimate *TART-A* copy number, we divided the median *TART-A* coverage of each strain by that strain’s median per-base coverage of all uniquely mappable positions in the *D*. *melanogaster* reference genome (calculated from the GC corrected, downsampled bam file). Uniquely mappable positions were identified using *mirth* (https://github.com/EvolBioInf/mirth).

We used the same pipeline to infer *TART-A* copy numbers for *D*. *simulans*. We used Illumina genomic sequencing data from [93] and, rather than aligning to RepBase TE consensus sequences, we identified TEs *de novo* using RepeatModeler (http://www.repeatmasker.org) with the long-read *D*. *simulans* genome assembly from [128]. The *D*. *simulans TART-A* sequence fragment is provided in (S4 Data).

### *Nxf2* expression in *D*. *simulans* versus *D*. *melanogaster* ovaries

For *D*. *simulans* strain *w501* and DGRP strains 313, 362, 379, 391, and 732, we used 5 to 20 pairs of ovaries from mated females. The ovaries were dissected in 1× PBS and then immediately transferred to 200-μL RNAlater Solution. Total RNA was extracted using 200-μL Trizol and DNase treated using the Ambion TURBO DNA-free Kit (Invitrogen, Carlsbad, California, USA). The mRNA-seq libraries were prepared using Bioo Scientific NEXTflex Poly(A) Beads and NEXTflex Rapid Directional RNA-Seq Kit (PerkinElmer, Austin, Texas, USA). We obtained additional *D*. *simulans* ovary expression data from [92]. We aligned the RNA-seq data to their respective reference genome assembly using hisat2 [115] and then used htseq-count [129] to obtain raw read counts for each gene. We only counted reads overlapping CDS features in case the UTR annotations differed between species. We corrected the raw counts for batch effects using ComBat-seq [130] and used DESeq2 to normalize the batch-corrected counts and test for differential expression. We only considered genes identified as 1-to-1 orthologs between *D*. *melanogaster* and *D*. *simulans* by FlyBase and excluded orthologs whose CDS length differed by more than 10 bp.

## Supporting information

S1 Fig Peptide alignment of Nxf2 homologs. We used NCBI web BLAST to search the *D*. *melanogaster* Nxf2 peptide sequence against the RefSeq peptide database and identified homologs in 22 *Drosophila* species. The carboxyl-terminal region of Nxf2 derives from CDS which shares homology with the *TART-A* TE (gray box). At the peptide level, this region is conserved out to *D*. *virilis*, which suggests that, if it was acquired from an insertion of the *TART-A* TE, the insertion would have occurred in the common ancestor of the entire genus. CDS, coding sequence; TE, transposable element.
(TIFF)

S2 Fig Zoom view of dotplot showing alignments of *D*. *melanogaster TART-A* versus *D*. *melanogaster nxf2* and *D*. *yakuba TART-A*. The pink boxes show the 2 segments of shared homology between *D*. *melanogaster TART-A* and *D*. *melanogaster nxf2*. *D*. *yakuba TART-A* aligns to *D*. *melanogaster TART-A* at regions directly adjacent to, but not including, the *TART-A/nxf2* shared homology. Underlying data can be found in S2 Data.
(TIFF)

S3 Fig Within-species comparisons of *nxf2* versus *TART-A*. We compared *nxf2* transcript sequences from *D*. *melanogaster* (A), *D*. *yakuba* (B), and *D*. *sechellia* (C) to *TART-A* sequences from the same species using *mummer* [106]. There is sequence homology present between *D*. *melanogaster nxf2* and *TART-A* but not *for D*. *yakuba nxf2/TART-A* nor for *D*. *sechellia nxf2/TART-A*. Underlying data can be found in S2 Data.
(TIFF)

S4 Fig Alignment of *nxf2*-like region from 71 *D*. *melanogaster TART-A* elements. We identified 71 *TART-A* elements with 3′ UTRs from 17 long-read *D*. *melanogaster* genome assemblies. All 71 elements contain the *nxf2*-like sequence (gray box) suggesting that this region is present in most, if not all, *TART-A* elements in *D*. *melanogaster*. Note that a portion of the *nxf2*-like region appears to have been deleted in one of the *TART-A* elements.
(TIFF)

S5 Fig Illumina sequencing coverage of the *nxf2*-like region of *TART-A* across the DGRP. We compared genomic sequencing coverage for the *nxf2*-like region of *TART-A* (blue shading) to its upstream and downstream flanking regions (yellow shading). For each DGRP strain, we divided read coverage by the median coverage of that strain’s *TART-A* ORF1 and ORF2 to control for copy number differences between strains. We calculated coverage for each strain in 10-bp windows across the region. Each box in the figure summarizes the per-strain coverage values for a single 10-bp segment. Within each box, the internal line represents the median coverage and the hinges correspond to the 25th and 75th percentiles. The whiskers extend to 1.5× the interquartile range. The coverage of the *nxf2*-like region is similar to the coverage of the downstream region, both of which are reduced relative to the upstream region. This pattern is consistent with truncation of the UTR, which has previously been described for *TART* [74]. Because the *nxf2*-like sequence is present in both UTRs, truncation of the 5′ UTR, which is fairly common, should reduce coverage of both the *nxf2*-like region and downstream flanking region by approximately 50% compared to the upstream region, which is not present in the 5′ UTR (Fig 1B). We observed a reduction in coverage of approximately 30%, consistent with a mixture of *TART-A* copies, some with truncated 5′ UTRs and some without. The median coverage across all boxes within a region is shown by the colored horizontal bars. Underlying data can be found in S2 Data. ORF, open reading frame.
(PDF)

S6 Fig Repetitive element up-regulation in *nxf2* knockdown. Each RepBase repeat for which we observed expression in total RNA-seq data from female ovaries is shown on the y-axis, and the fold change in expression in the *nxf2* RNAi knockdown versus a control knockdown of the *white* gene is shown on the x-axis with a log2 scale. Expression values are the mean of 2 biological replicates for both knockdown and control. For LTR retrotransposons, LTRs are shown separately from the rest of the TE. Underlying data can be found in S2 Data. LTR, long terminal repeat; TE, transposable element.
(PDF)

S7 Fig Correlation between shRNAs in *nxf2* knockdown. We used 2 shRNAs that target different regions of the *nxf2* transcript and calculated expression values for genes as well as TEs for each knockdown. We found that the expression values are highly correlated between the 2 experiments (Spearman’s rho = 0.92 [Genes] and 0.94 [TEs]). Underlying data can be found in S2 Data. shRNA, short hairpin RNA; TE, transposable element.
(PDF)

S8 Fig *nxf2* cleavage products from degradome-seq data. We analyzed published degradome-seq and Aub-immunoprecipitated small RNA data to determine whether there were *nxf2* degradome-seq reads showing the 10-bp sense:antisense overlap with *TART-A* piRNAs, consistent with cleavage by a Piwi protein. We identified 11 locations (A–K) within the *TART*-like region of *nxf2* where degradome-seq cleavage products (red) overlap with antisense piRNAs (blue) by 10 bp at their 5′ ends. The *nxf2* transcript is shown in black. degradome-seq, degradome sequencing; piRNA, Piwi-interacting small RNA.
(TIFF)

S9 Fig Genes up-regulated upon disruption of the piRNA pathway show greater abundance of aligned piRNAs. We identified 168 genes whose fold change in expression was greater than or equal to *nxf2* across RNAi knockdowns of 16 piRNA pathway components. These genes have a significantly larger abundance of aligned piRNAs compared to the remainder of expressed genes, suggesting their expression may be regulated by piRNAs (Wilcoxon test *P* = 4.1e-06). Underlying data can be found in S2 Data. piRNA, Piwi-interacting small RNA; RNAi, RNA interference.
(PDF)

S10 Fig PiRNA pathway genes do not show a uniform response to piRNA pathway disruption. We examined the fold change in expression of 41 known piRNA pathway genes across RNAi knockdowns of 16 piRNA pathway components, excluding the targeted gene from analysis for each experiment. PiRNA pathway genes show a median fold change near 1 (horizontal red line) for most experiments. Underlying data can be found in S2 Data. piRNA, Piwi-interacting small RNA; RNAi, RNA interference.
(PDF)

S11 Fig The correlation between *nxf2* expression and *TART-A* copy number is reproducible. We repeated the analysis shown in Fig 7A using a replicate microarray dataset from [125] and found a similar correlation (Spearman’s rho = −0.49), which suggests that the microarray expression measurements are highly reproducible. Underlying data can be found in S2 Data.
(PDF)

S12 Fig Expression of other piRNA pathway genes (besides *nxf2*) is not correlated with *TART-A* copy number. We were able to obtain expression values for 39 other piRNA pathway genes from the same microarray dataset that we used for *nxf2* expression. For each of these genes, we calculated Spearman correlation coefficient for its expression compared to *TART-A* copy number. All correlation coefficients are at least 2-fold smaller in magnitude than what we observed for nxf2. Underlying data can be found in S2 Data. piRNA, Piwi-interacting small RNA.
(TIFF)

S13 Fig Summary of correlations between piRNA pathway genes and *TART-A* copy number. The histogram summarizes the Spearman correlation coefficients between 39 piRNA pathway genes and *TART-A* copy number (shown in S12 Fig). The red line shows the correlation coefficient for *nxf2*. Underlying data can be found in S2 Data. piRNA, Piwi-interacting small RNA.
(PDF)

S14 Fig Per-strain piRNA coverage of *nxf2*. We plotted piRNA read depth (normalized as RPM mapped) along the *nxf2* transcript for each of the 16 DGRP strains shown in Fig 7. For each strain, the abundance of *TART* piRNAs is listed in the plot title. We masked the locations of the *TART*/*nxf2* shared homology (gray boxes) before alignment to avoid cross-mapping of *TART*-derived piRNAs. Underlying data can be found in S2 Data. DGRP, *Drosophila* Genetic Reference Panel; piRNA, Piwi-interacting small RNA; RPM, reads per million.
(PDF)

S15 Fig Correlation between *TART-A* and *nxf2* piRNAs. There is a strong positive correlation between *TART*-derived piRNAs that align to *nxf2* versus the *nxf2* piRNAs downstream from the region of shared homology, across 16 DGRP strains (Spearman’s rho = 0.88, *P* < 2.2e-16). Underlying data can be found in S2 Data. DGRP, *Drosophila* Genetic Reference Panel; piRNA, Piwi-interacting small RNA.
(PDF)

S16 Fig The 5 DGRP strains used in the RNA-seq experiment have *nxf2* expression levels that are representative of the DGRP population as a whole. We used the microarray dataset from [125] to select 5 DGRP strains whose median *nxf2* expression level is similar to that of the full DGRP population. Underlying data can be found in S2 Data. DGRP, *Drosophila* Genetic Reference Panel; RNA-seq, RNA sequencing.
(TIFF)

S1 Table Allele-specific counts for *TART*-derived antisense piRNAs aligned to *nxf2*. piRNA, Piwi-interacting small RNA. (DOCX)

S1 Data Multiple sequence alignment of *nxf2*. (XLSX)

S2 Data Underlying data for all graphs. (XLSX)

S3 Data Multiple sequence alignment used for Fig 2B. (TXT)

S4 Data FASTA file containing the sequence of the *D*. *simulans TART-A* fragment. (TXT)

## Abbreviations

CDS: coding sequence
degradome-seq: degradome sequencing
DGRP: *Drosophila* Genetic Reference Panel
HTT: *HeT-A*, *TAHRE*, and *TART*
LINE: Long Interspersed Nuclear Element
LTR: long terminal repeat
mRNA-seq: mRNA sequencing
ORF: open reading frame
piRNA: Piwi-interacting small RNA
RNAi: RNA interference
RNA-seq: RNA sequencing
RPM: reads per million
shRNA: short hairpin RNA
TE: transposable element
TRiP: Transgenic RNAi Project
