# Supplementary figures and images for: Telomeric TART elements target the piRNA machinery in Drosophila
Source: PLoS Biol. 2020 Dec 21;18(12):e3000689. doi: 10.1371/journal.pbio.3000689 (PMC7785250; doi:10.1371/journal.pbio.3000689)

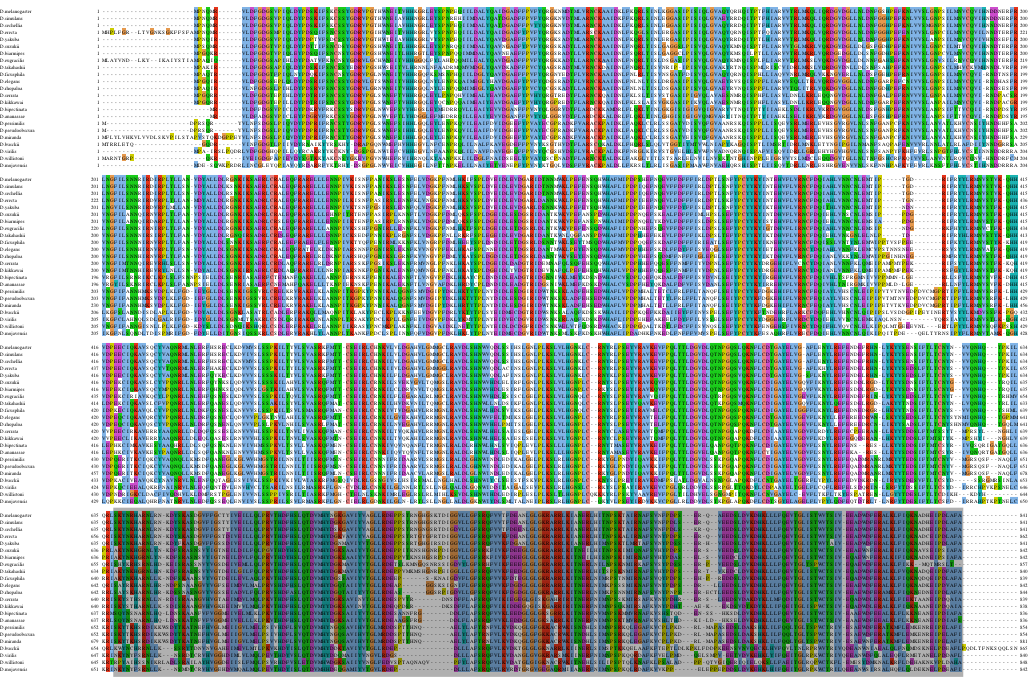

Supplement: S1 Fig — We used NCBI web BLAST to search the D. melanogaster Nxf2 peptide sequence against the RefSeq peptide database and identified homologs in 22 Drosophila species. The carboxyl-terminal region of Nxf2 derives from CDS which shares homology with the TART-A TE (gray box). At the peptide level, this region is conserved out to D. virilis, which suggests that, if it was acquired from an insertion of the TART-A TE, the insertion would have occurred in the common ancestor of the entire genus. CDS, coding sequence; TE, transposable element. (TIFF) [file pbio.3000689.s001.tiff]

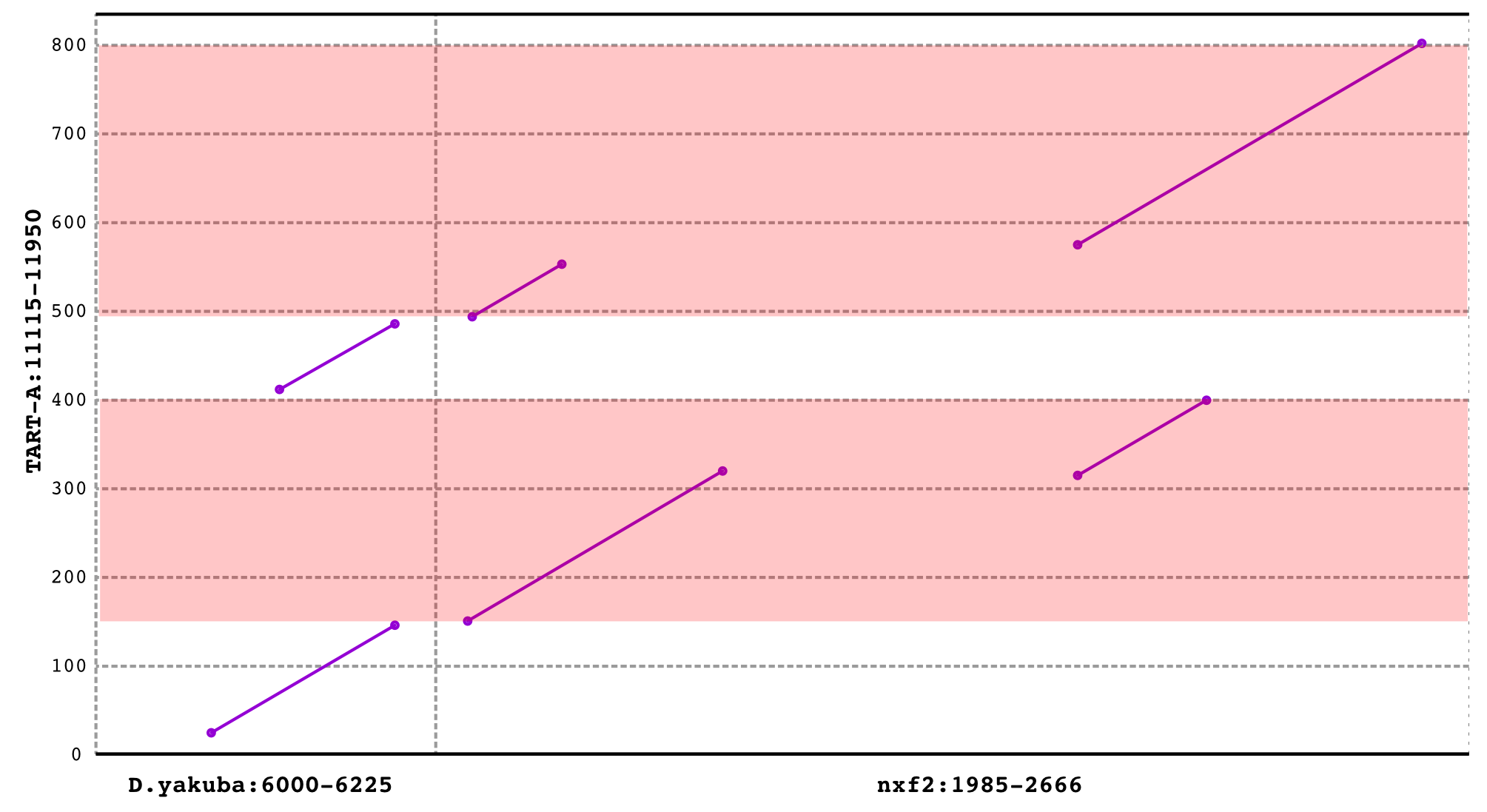

Supplement: S2 Fig — The pink boxes show the 2 segments of shared homology between D. melanogaster TART-A and D. melanogaster nxf2. D. yakuba TART-A aligns to D. melanogaster TART-A at regions directly adjacent to, but not including, the TART-A/nxf2 shared homology. Underlying data can be found in S2 Data. (TIFF) [file pbio.3000689.s002.tiff]

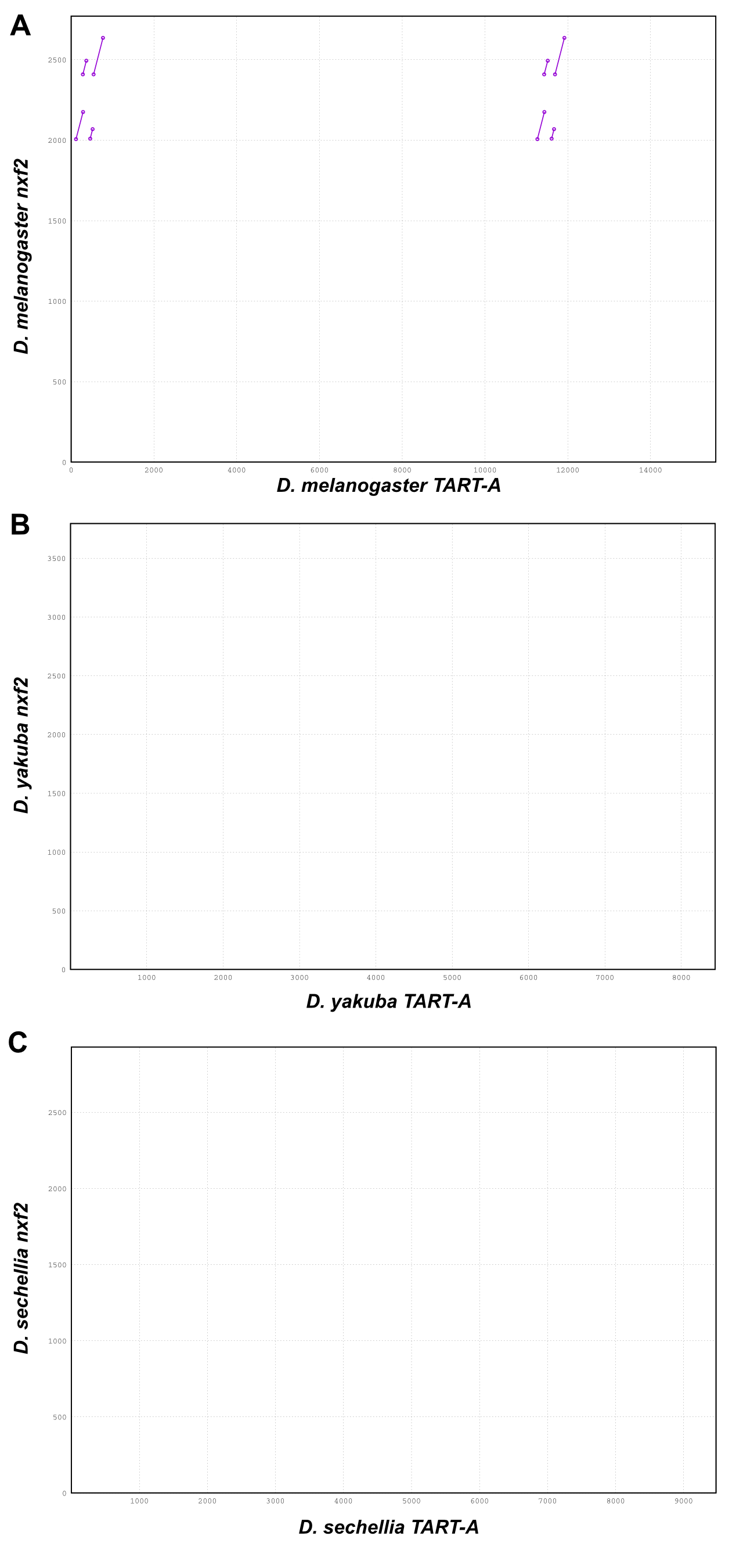

Supplement: S3 Fig — We compared nxf2 transcript sequences from D. melanogaster (A), D. yakuba (B), and D. sechellia (C) to TART-A sequences from the same species using mummer [106]. There is sequence homology present between D. melanogaster nxf2 and TART-A but not for D. yakuba nxf2/TART-A nor for D. sechellia nxf2/TART-A. Underlying data can be found in S2 Data. (TIFF) [file pbio.3000689.s003.tiff]

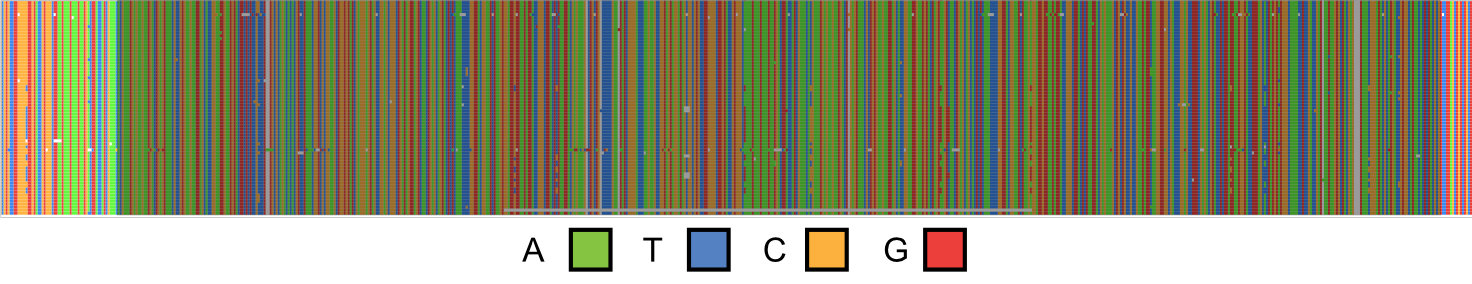

Supplement: S4 Fig — We identified 71 TART-A elements with 3′ UTRs from 17 long-read D. melanogaster genome assemblies. All 71 elements contain the nxf2-like sequence (gray box) suggesting that this region is present in most, if not all, TART-A elements in D. melanogaster. Note that a portion of the nxf2-like region appears to have been deleted in one of the TART-A elements. (TIFF) [file pbio.3000689.s004.tiff]

Normalized Coverage

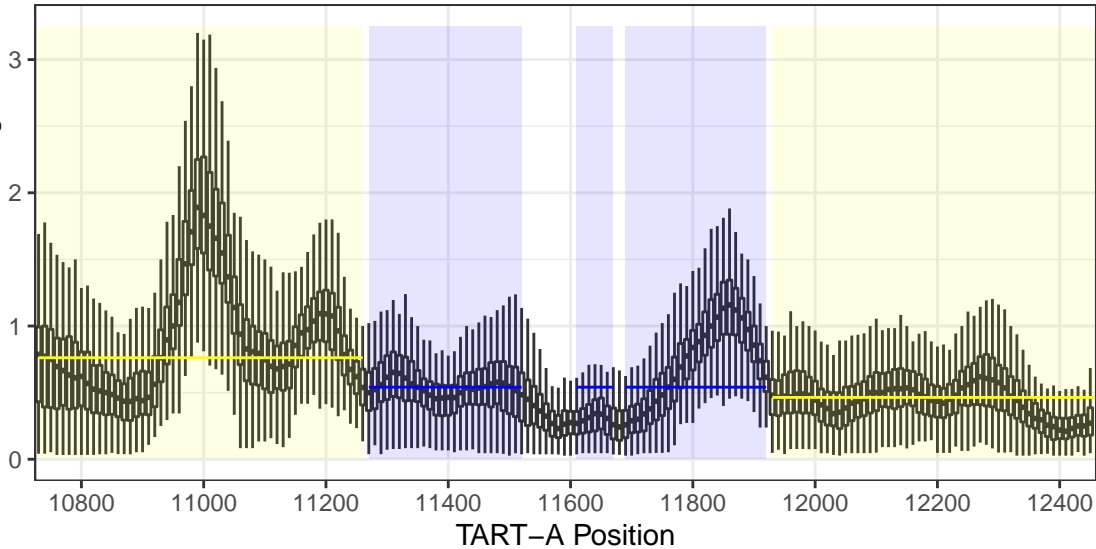

Supplement: S5 Fig — We compared genomic sequencing coverage for the nxf2-like region of TART-A (blue shading) to its upstream and downstream flanking regions (yellow shading). For each DGRP strain, we divided read coverage by the median coverage of that strain’s TART-A ORF1 and ORF2 to control for copy number differences between strains. We calculated coverage for each strain in 10-bp windows across the region. Each box in the figure summarizes the per-strain coverage values for a single 10-bp segment. Within each box, the internal line represents the median coverage and the hinges correspond to the 25th and 75th percentiles. The whiskers extend to 1.5× the interquartile range. The coverage of the nxf2-like region is similar to the coverage of the downstream region, both of which are reduced relative to the upstream region. This pattern is consistent with truncation of the UTR, which has previously been described for TART [74]. Because the nxf2-like sequence is present in both UTRs, truncation of the 5′ UTR, which is fairly common, should reduce coverage of both the nxf2-like region and downstream flanking region by approximately 50% compared to the upstream region, which is not present in the 5′ UTR (Fig 1B). We observed a reduction in coverage of approximately 30%, consistent with a mixture of TART-A copies, some with truncated 5′ UTRs and some without. The median coverage across all boxes within a region is shown by the colored horizontal bars. Underlying data can be found in S2 Data. ORF, open reading frame. (PDF) [file pbio.3000689.s005.pdf]

# TE Family

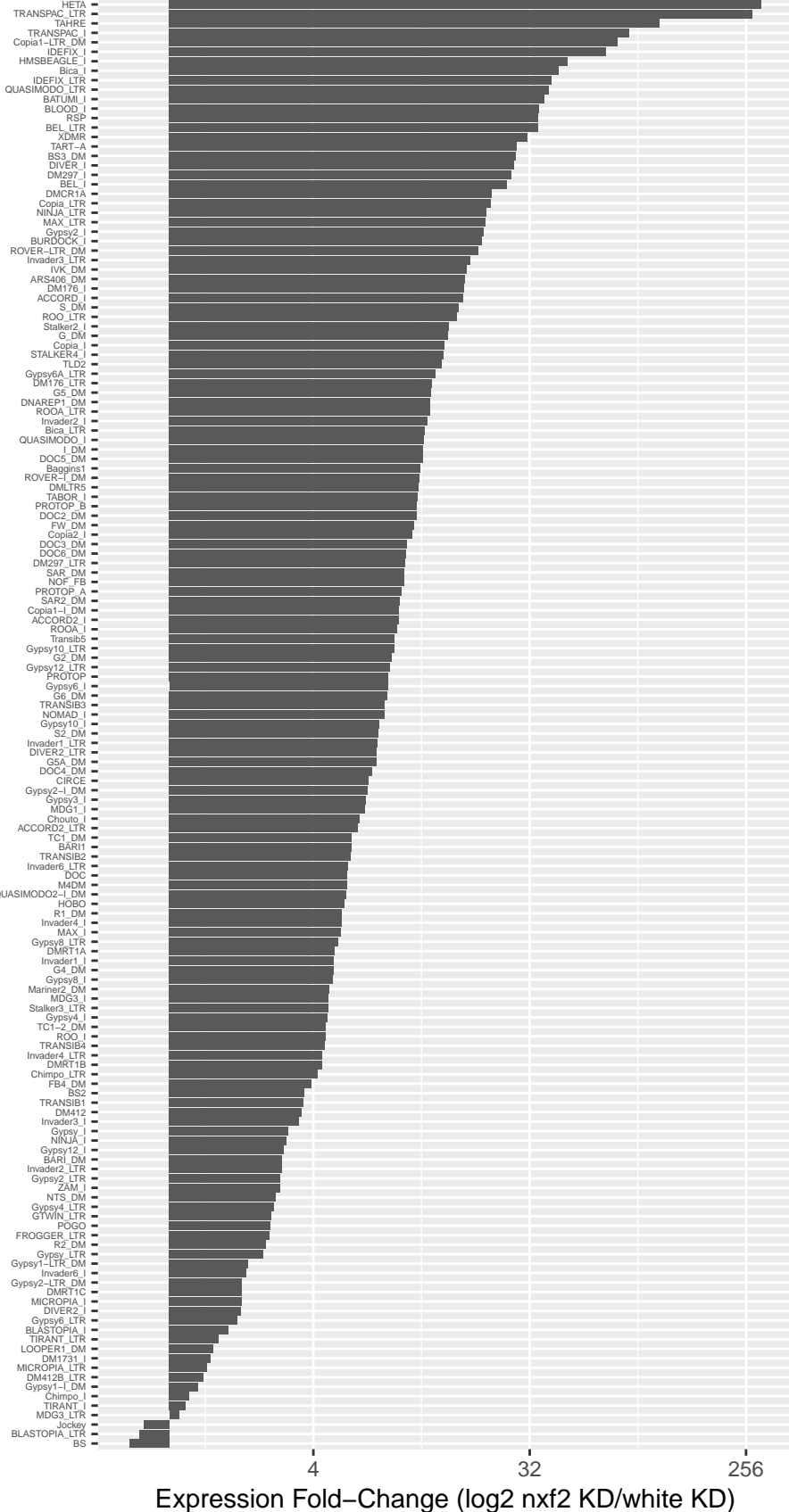

Supplement: S6 Fig — Each RepBase repeat for which we observed expression in total RNA-seq data from female ovaries is shown on the y-axis, and the fold change in expression in the nxf2 RNAi knockdown versus a control knockdown of the white gene is shown on the x-axis with a log2 scale. Expression values are the mean of 2 biological replicates for both knockdown and control. For LTR retrotransposons, LTRs are shown separately from the rest of the TE. Underlying data can be found in S2 Data. LTR, long terminal repeat; TE, transposable element. (PDF) [file pbio.3000689.s006.pdf]

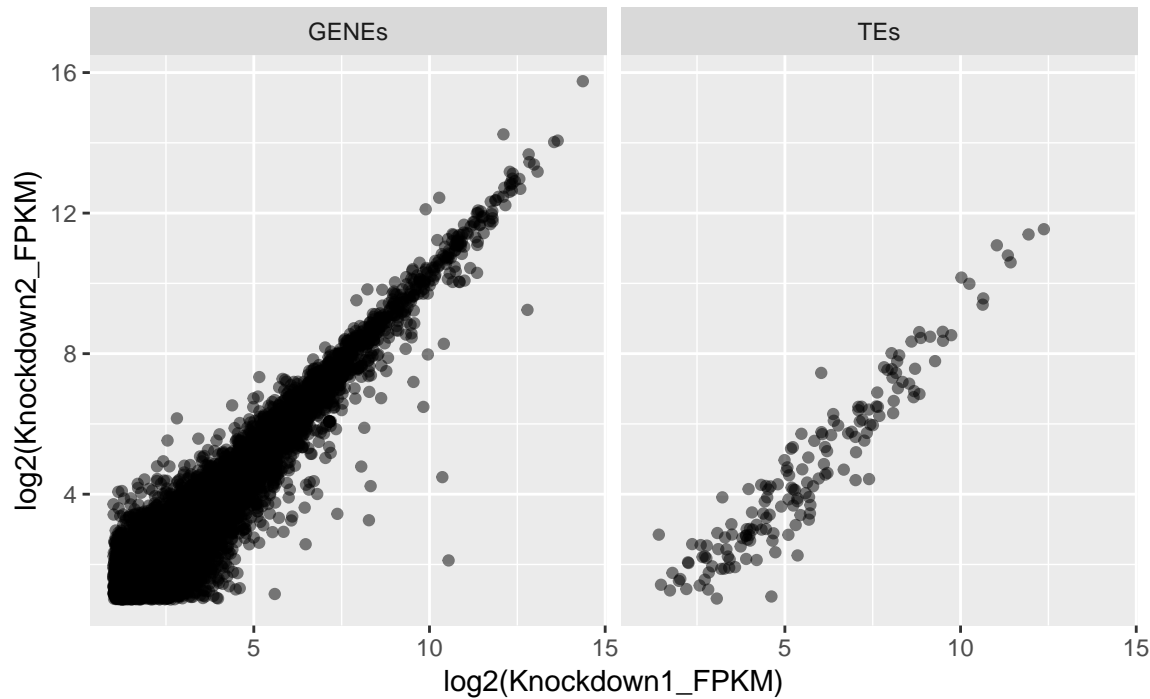

Supplement: S7 Fig — We used 2 shRNAs that target different regions of the nxf2 transcript and calculated expression values for genes as well as TEs for each knockdown. We found that the expression values are highly correlated between the 2 experiments (Spearman’s rho = 0.92 [Genes] and 0.94 [TEs]). Underlying data can be found in S2 Data. shRNA, short hairpin RNA; TE, transposable element. (PDF) [file pbio.3000689.s007.pdf]

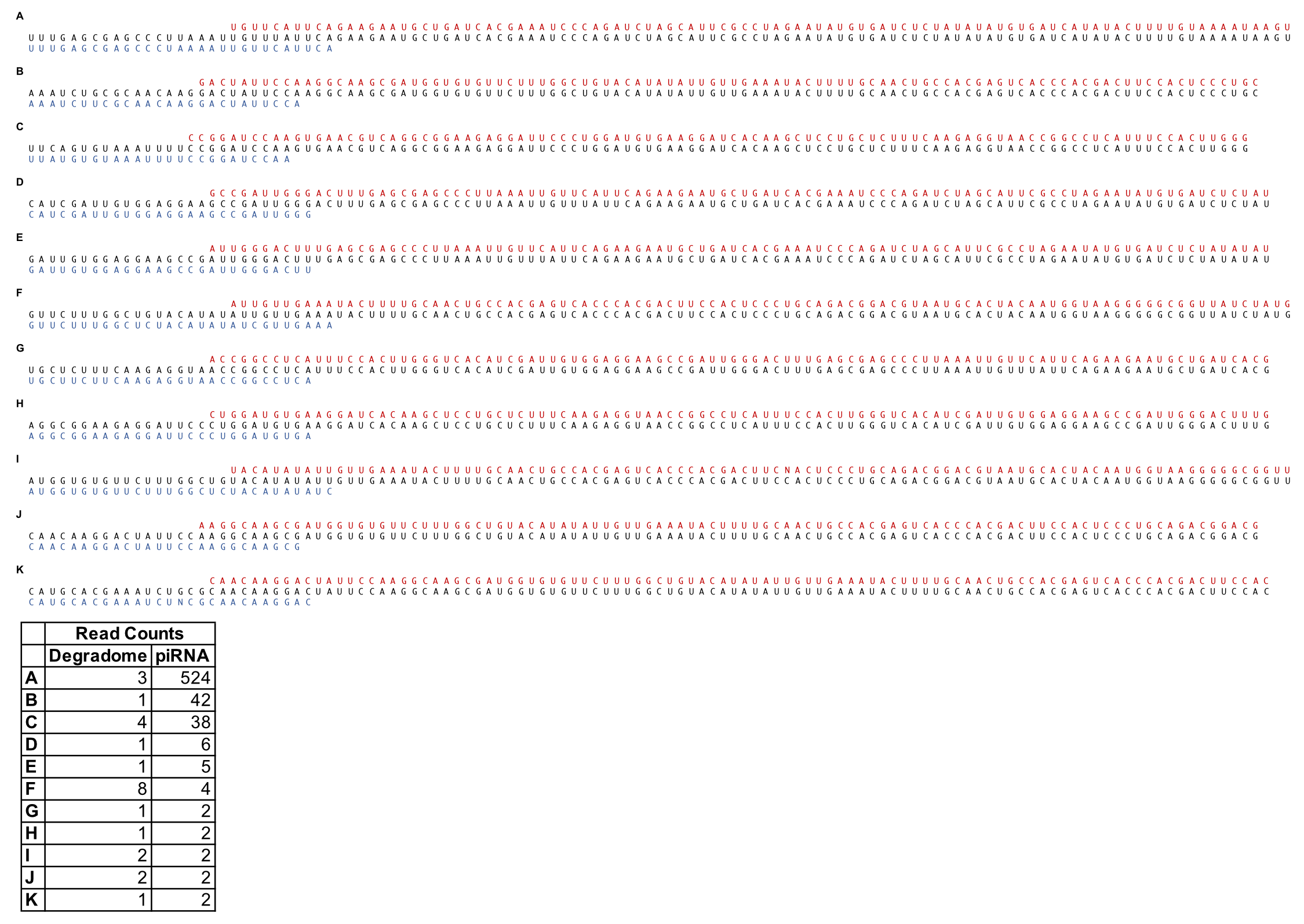

Supplement: S8 Fig — We analyzed published degradome-seq and Aub-immunoprecipitated small RNA data to determine whether there were nxf2 degradome-seq reads showing the 10-bp sense:antisense overlap with TART-A piRNAs, consistent with cleavage by a Piwi protein. We identified 11 locations (A–K) within the TART-like region of nxf2 where degradome-seq cleavage products (red) overlap with antisense piRNAs (blue) by 10 bp at their 5′ ends. The nxf2 transcript is shown in black. degradome-seq, degradome sequencing; piRNA, Piwi-interacting small RNA. (TIFF) [file pbio.3000689.s008.tiff]

piRNAs aligned to gene: log2(FPKM)

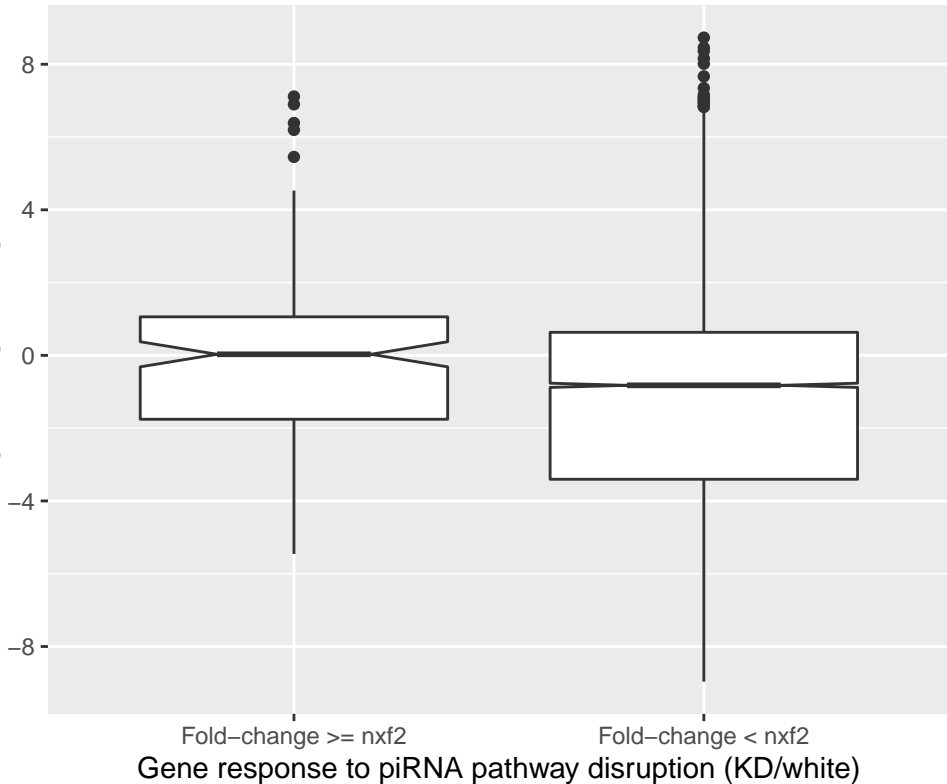

Supplement: S9 Fig — We identified 168 genes whose fold change in expression was greater than or equal to nxf2 across RNAi knockdowns of 16 piRNA pathway components. These genes have a significantly larger abundance of aligned piRNAs compared to the remainder of expressed genes, suggesting their expression may be regulated by piRNAs (Wilcoxon test P = 4.1e-06). Underlying data can be found in S2 Data. piRNA, Piwi-interacting small RNA; RNAi, RNA interference. (PDF) [file pbio.3000689.s009.pdf]

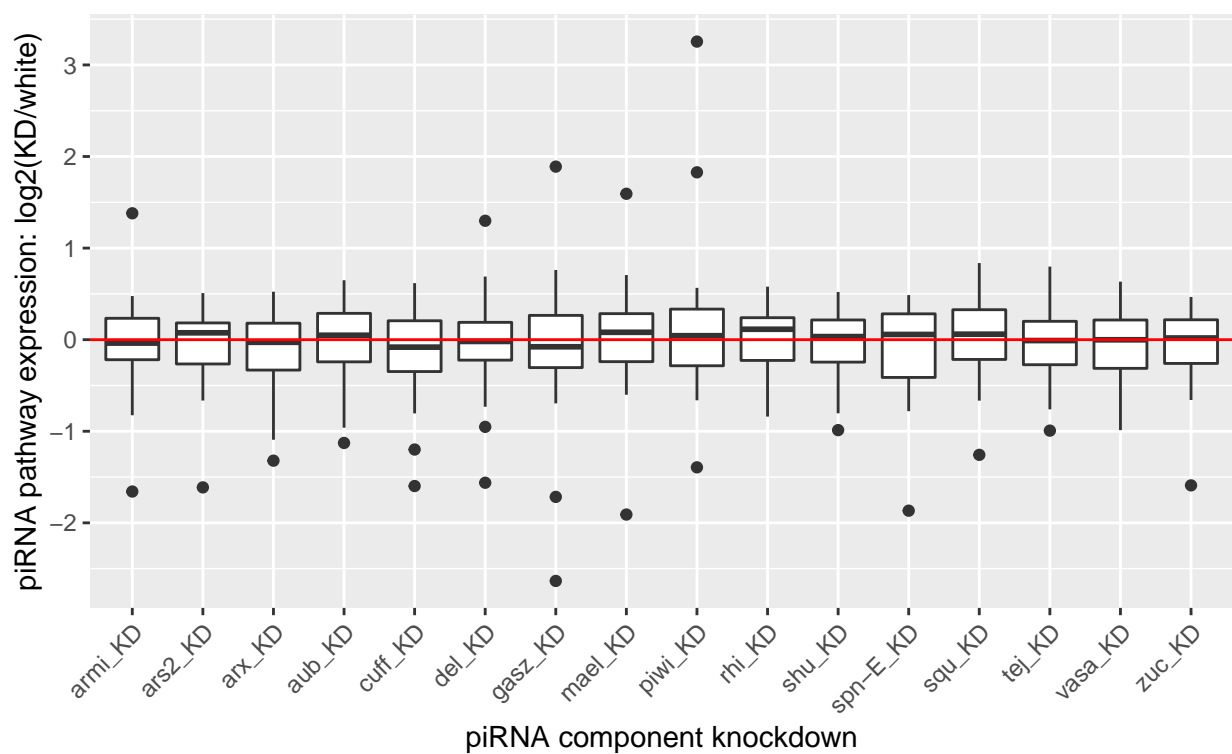

Supplement: S10 Fig — We examined the fold change in expression of 41 known piRNA pathway genes across RNAi knockdowns of 16 piRNA pathway components, excluding the targeted gene from analysis for each experiment. PiRNA pathway genes show a median fold change near 1 (horizontal red line) for most experiments. Underlying data can be found in S2 Data. piRNA, Piwi-interacting small RNA; RNAi, RNA interference. (PDF) [file pbio.3000689.s010.pdf]

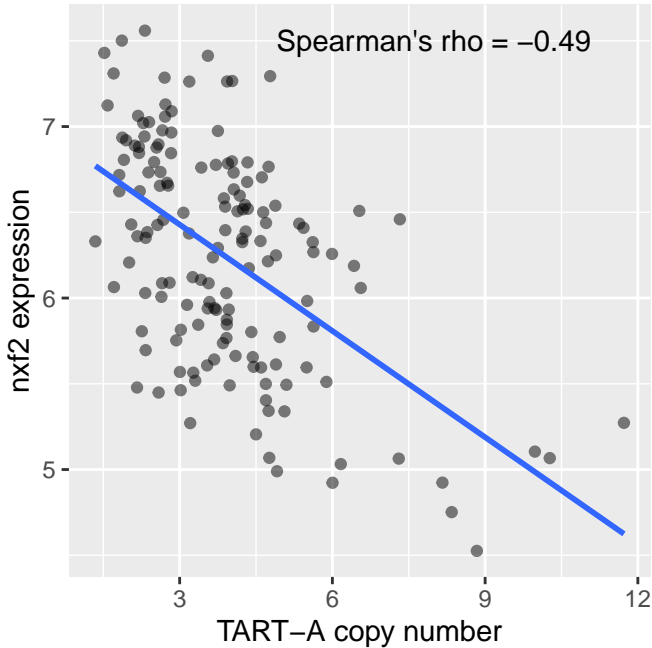

Supplement: S11 Fig — We repeated the analysis shown in Fig 7A using a replicate microarray dataset from [125] and found a similar correlation (Spearman’s rho = −0.49), which suggests that the microarray expression measurements are highly reproducible. Underlying data can be found in S2 Data. (PDF) [file pbio.3000689.s011.pdf]

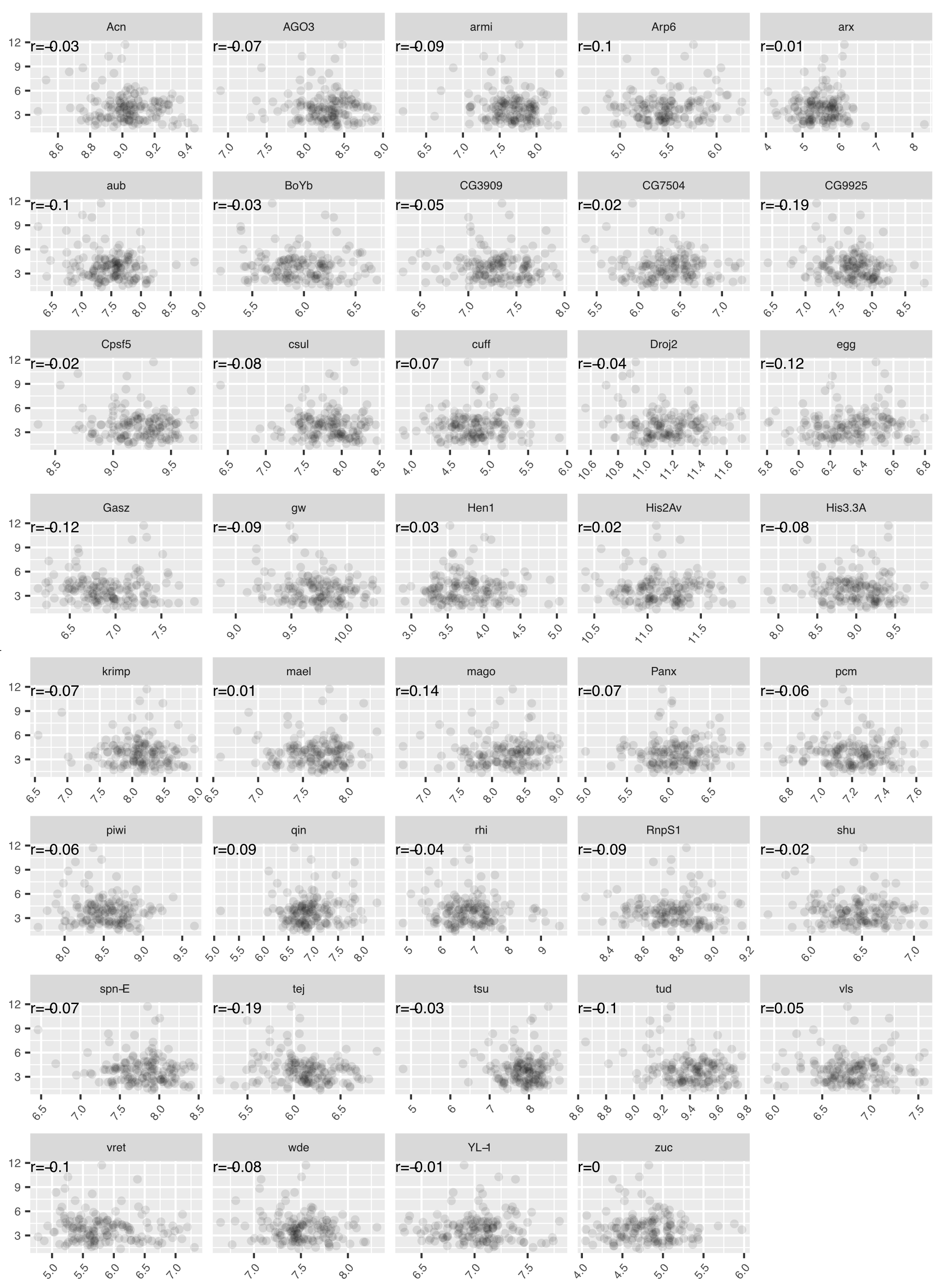

Supplement: S12 Fig — We were able to obtain expression values for 39 other piRNA pathway genes from the same microarray dataset that we used for nxf2 expression. For each of these genes, we calculated Spearman correlation coefficient for its expression compared to TART-A copy number. All correlation coefficients are at least 2-fold smaller in magnitude than what we observed for nxf2. Underlying data can be found in S2 Data. piRNA, Piwi-interacting small RNA. (TIFF) [file pbio.3000689.s012.tiff]

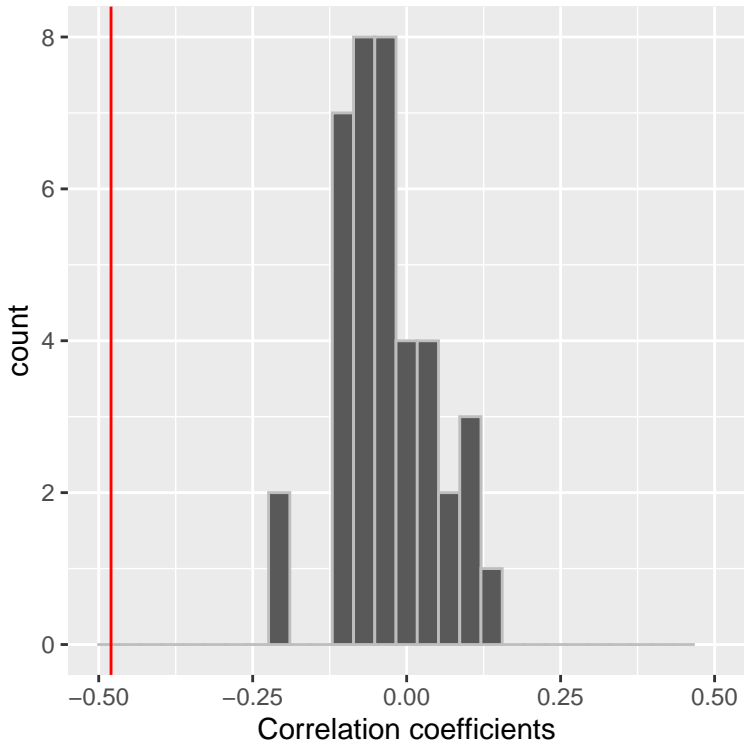

Supplement: S13 Fig — The histogram summarizes the Spearman correlation coefficients between 39 piRNA pathway genes and TART-A copy number (shown in S12 Fig). The red line shows the correlation coefficient for nxf2. Underlying data can be found in S2 Data. piRNA, Piwi-interacting small RNA. (PDF) [file pbio.3000689.s013.pdf]

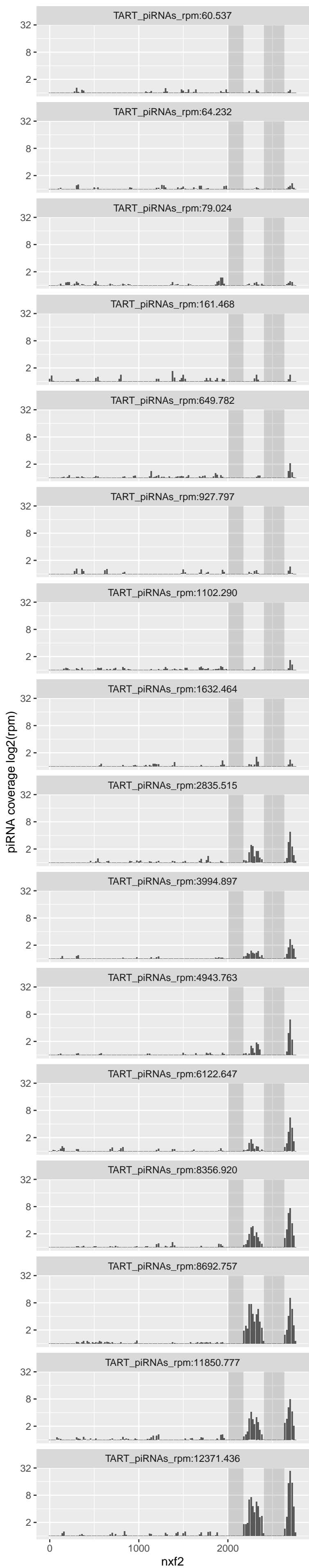

Supplement: S14 Fig — We plotted piRNA read depth (normalized as RPM mapped) along the nxf2 transcript for each of the 16 DGRP strains shown in Fig 7. For each strain, the abundance of TART piRNAs is listed in the plot title. We masked the locations of the TART/nxf2 shared homology (gray boxes) before alignment to avoid cross-mapping of TART-derived piRNAs. Underlying data can be found in S2 Data. DGRP, Drosophila Genetic Reference Panel; piRNA, Piwi-interacting small RNA; RPM, reads per million. (PDF) [file pbio.3000689.s014.pdf]

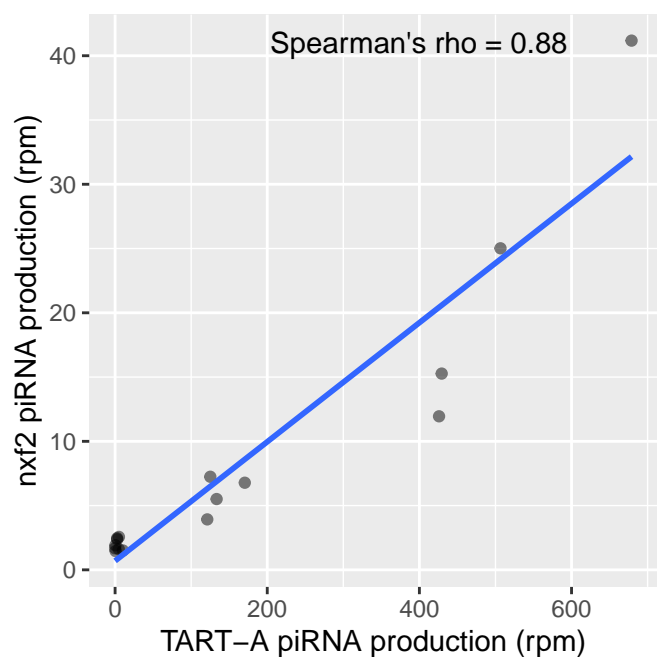

Supplement: S15 Fig — There is a strong positive correlation between TART-derived piRNAs that align to nxf2 versus the nxf2 piRNAs downstream from the region of shared homology, across 16 DGRP strains (Spearman’s rho = 0.88, P < 2.2e-16). Underlying data can be found in S2 Data. DGRP, Drosophila Genetic Reference Panel; piRNA, Piwi-interacting small RNA. (PDF) [file pbio.3000689.s015.pdf]

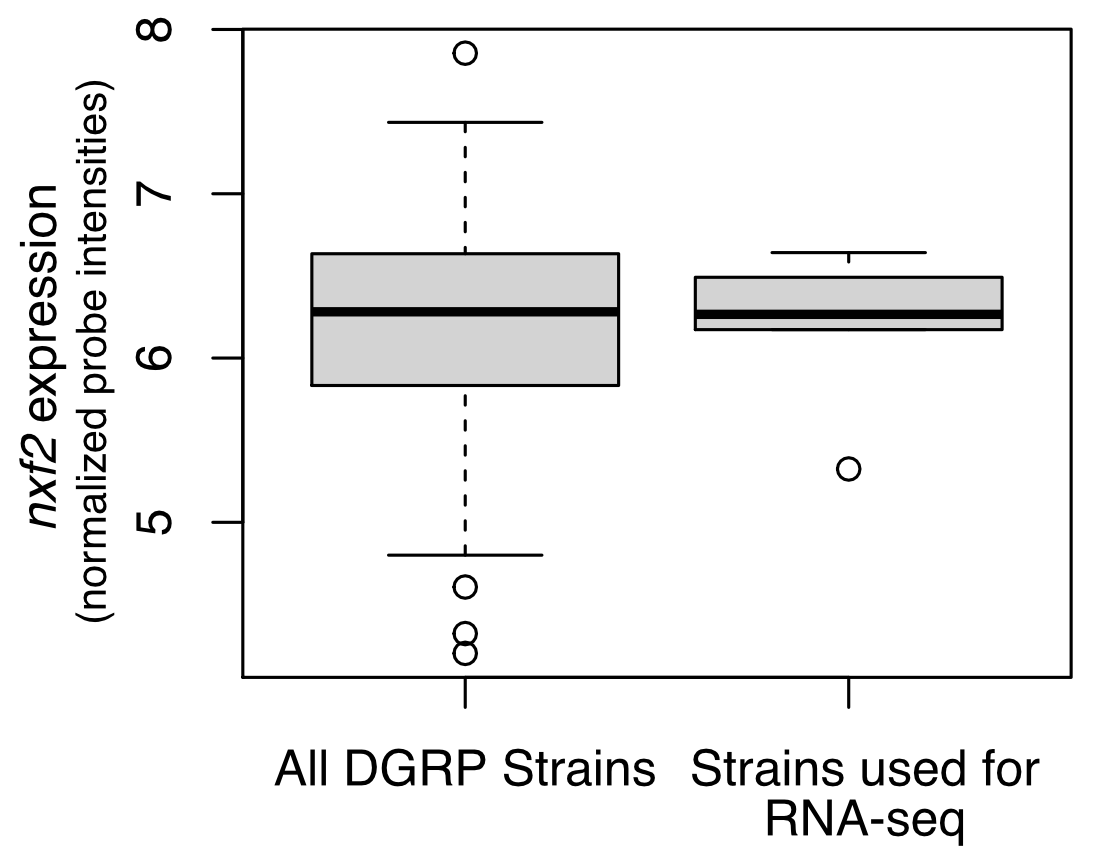

Supplement: S16 Fig — We used the microarray dataset from [125] to select 5 DGRP strains whose median nxf2 expression level is similar to that of the full DGRP population. Underlying data can be found in S2 Data. DGRP, Drosophila Genetic Reference Panel; RNA-seq, RNA sequencing. (TIFF) [file pbio.3000689.s016.tiff]
