# Supplementary material for: Telomeric TART elements target the piRNA machinery in Drosophila
Source: PLoS Biol. 2020 Dec 21;18(12):e3000689. doi: 10.1371/journal.pbio.3000689 (PMC7785250; doi:10.1371/journal.pbio.3000689)
Supplement: S1 Table — (DOCX) [file pbio.3000689.s017.docx]

|  | **Allele-specific counts of antisense piRNA bases aligned to *nxf2*** | | |
| --- | --- | --- | --- |
| ***nxf2* position** | ***TART-A*** | ***nxf2*** | **Other** |
| 2015 | 175 | 1 | 0 |
| 2022 | 198 | 1 | 1 |
| 2085 | 2417 | 2 | 5 |
| 2095 | 164 | 0 | 4 |
| 2104 | 0 | 4 | 0 |
| 2108 | 12 | 2 | 0 |
| 2110 | 64 | 0 | 3 |
| 2119 | 173 | 0 | 0 |
| 2168 | 34 | 0 | 1 |
| 2172 | 2 | 0 | 0 |
| 2173 | 2 | 0 | 0 |
| 2476 | 69 | 0 | 1 |
| 2479 | 73 | 0 | 0 |
| 2485 | 79 | 0 | 0 |
| 2566 | 2143 | 0 | 3 |
| 2575 | 2685 | 10 | 5 |
| 2587 | 954 | 7 | 7 |
